# Follow the Trail: Machine Learning for Fraud Detection in Fintech Applications

**DOI:** 10.3390/s21051594

**Published:** 2021-02-25

**Authors:** Branka Stojanović, Josip Božić, Katharina Hofer-Schmitz, Kai Nahrgang, Andreas Weber, Atta Badii, Maheshkumar Sundaram, Elliot Jordan, Joel Runevic

**Affiliations:** 1 Joanneum Research, DIGITAL—Institute for Information and Communication Technologies, A-8010 Graz, Austria; josip.bozic@joanneum.at (J.B.); katharina.hofer-schmitz@joanneum.at (K.H.-S.); kai.nahrgang@joanneum.at (K.N.); 2 Fraunhofer Institute for High-Speed Dynamics, Ernst-Mach-Institut, EMI, D-79588 Efringen-Kirchen, Germany; andreas.weber@emi.fraunhofer.de; 3 Department of Computer Science, School of Mathematical, Physical and Computational Sciences, University of Reading, Reading RG6 6AH, UK; atta.badii@reading.ac.uk (A.B.); m.sundaram@reading.ac.uk (M.S.); elliotjacob.jordan@reading.ac.uk (E.J.); j.runevic@reading.ac.uk (J.R.)

**Keywords:** fraud detection, machine learning, anomaly detection, Fintech, cybercrime

## Abstract

Financial technology, or Fintech, represents an emerging industry on the global market. With online transactions on the rise, the use of IT for automation of financial services is of increasing importance. Fintech enables institutions to deliver services to customers worldwide on a 24/7 basis. Its services are often easy to access and enable customers to perform transactions in real-time. In fact, advantages such as these make Fintech increasingly popular among clients. However, since Fintech transactions are made up of information, ensuring security becomes a critical issue. Vulnerabilities in such systems leave them exposed to fraudulent acts, which cause severe damage to clients and providers alike. For this reason, techniques from the area of Machine Learning (ML) are applied to identify anomalies in Fintech applications. They target suspicious activity in financial datasets and generate models in order to anticipate future frauds. We contribute to this important issue and provide an evaluation on anomaly detection methods for this matter. Experiments were conducted on several fraudulent datasets from real-world and synthetic databases, respectively. The obtained results confirm that ML methods contribute to fraud detection with varying success. Therefore, we discuss the effectiveness of the individual methods with regard to the detection rate. In addition, we provide an analysis on the influence of selected features on their performance. Finally, we discuss the impact of the observed results for the security of Fintech applications in the future.

## 1. Introduction

Modern-day demands for services require availability and worldwide accessibility around-the-clock. Fintech represents the application of IT solutions in business models in order to deliver improved financial services to clients. The term itself, however, is still much under debate. In fact, Fintech represents a blanket term for a broad scope of technologies that dynamically interact in a common infrastructure. The word Fintech was initially coined in 1972, when it was defined as “an acronym which stands for financial technology, combining bank expertise with modern management science techniques and the computer” [1]. Since then, the term has stood for the continuous co-evolution of technology and finance. Companies that apply this business model offer advantages, such as easier use and cheaper and more secure transactions [2]. Fintech services have become more attractive for both clients and providers. This fact is further confirmed by the constant rise of Fintech investments over the last few years [2,3]. In the future, Fintech might outperform and even replace traditional finance institutions.

In practice, Fintech relies on different payment methods, such as credit cards and financial transactions, which include digital currencies. The latter is built upon the technological foundation of Blockchain, which provides a direct connection to financial institutions. In Fintech, financial transactions represent repetitive processes where sensitive information is exchanged between two peers. Existing AI technologies often supplement Fintech services by executing these processes in an automated and secure manner. Besides functionality, however, business models that are used for Fintech must ensure information security. Since the Fintech business model relies on an existing IT infrastructure, financial activities are subject to exploitation. In general, fraudulent acts target specific weaknesses in financial activities. These include, among others, credit cards, financial transactions and the underlying Blockchain technology. Malicious activity is executed by single or multiple perpetrators and can lead to severe consequences. To add weight to this fact, only a minority of organisations implement any anti-fraud mechanisms [4]. In the aftermath, just a small minority of fraud victims ever fully recover [5].

The identification of such malicious acts represents a major technical challenge for the Fintech industry. For this reason, intelligent approaches from the domain of machine learning (ML) are applied in order to detect suspicious fraudulent patterns. ML encompasses anomaly detection techniques that automatically identify and classify suspicious data from financial networks. Methods such as learning algorithms, statistical models and artificial neural networks (ANN) are used to generate models from a dataset. Next, the resulting representation is observed in order to derive appropriate fraud prevention techniques and policies.

It should be noted that ML methods come with certain challenges and trade-offs in real-world implementations. The most common issue is the number of false positives—if it is high it results in a large number of false alerts and subsequently with service provider work overload and customer dissatisfaction.

In this paper, an overview of existing ML methods is provided for fraud detection in the Fintech domain. The available anomaly detection techniques are also discussed with regard to efficiency in fraud detection. The application of ML methods is then demonstrated in multiple case studies. Subsequently, the empirical results obtained from a fraudulent dataset for the banking domain are explained. Finally, the implications of the methodology on fraud detection for the future are set out.

The remainder of the paper is structured as follows. Section 2 provides an overview about existing literature on ML methods for fraud detection. Section 3 describes the underlying methods and models in detail. Section 4 discusses the evaluation methodology, which is demonstrated in several case studies in Section 5. Section 6 discusses non-technological aspects of the discussed technology. Section 7 concludes the paper.

## 2. Fraud Detection in the Fintech Domain

First, a basic definition of frauds in the context of Fintech is explained. In theory, a fraud can be defined as “an intentionally deceptive action designed to provide the perpetrator with an unlawful gain or to deny a right to a victim” [6]. Unfortunately, detecting frauds represents a major challenge since fraud is an adaptive crime [7], hence the need for large scale financial datasets. A dataset represents an aggregation of transactions from the financial network traffic during a specific period. In such datasets, a fraud manifests itself as an anomaly that differs from the usual records [8]. In order to identify such patterns, techniques were applied to anomaly detection in the domain of ML. In general, anomaly detection techniques enable the identification of frauds from large datasets. They have been proven to achieve good results in classifying unusual data in such aggregations. In fact, these advantages make them a natural choice to address fraud detection challenges. In this paper, three different types of frauds are focused on, namely in credit cards, financial and blockchain transactions. A description about the used anomaly detection techniques is given in Section 3.

Unfortunately, a general problem for the research in anomaly detection in Fintech is the absence of publicly available test data. Therefore, the most known accessible and widely used data represent the Kaggle datasets. These include datasets for credit cards [9], bank transaction data [10] and blockchain historical data [11]. Other known, slightly older, synthetic datasets can be found in the UC Irvine ML Repository (e.g., UC Irvine [12]). In addition, simulators such as BankSim [13] and PaySim [14] are applied to address this problem. The former represents an agent-based simulator of bank payments, whereas the latter simulates mobile transactions by generating clients and performing transactions. In both cases, the result represents a dataset that resembles real users and transactions. Many of the related papers below rely on these datasets for anomaly detection.

Besides the absence of publicly available datasets, there are two other main challenges in the field of fraud detection: the class imbalance, i.e., there are much more genuine transactions than fraudulent transactions, and the concept drift, i.e., the habits of customers and fraudsters evolve [15,16]. There are several approaches which focus on concept drift. Dal Pozzolo et al. [15] designed two fraud-detection systems based on ensemble and a sliding-window approach for a concept-drift adaptation. In newer work by the same group of authors [16], concept drift is also taken into account. The concept drift aspect is also in focus in the work by Ma et al. [17], where an incremental virtual learning method for the update of neural networks is proposed. Somasundaram and Reddy [18] have proposed a parallel and incremental learning ensemble to deal with the concept drift and data imbalance.

There are several survey papers covering anomaly detection in Fintech, which provide a very good insight into current trends. An early comprehensive study of intelligent solutions for financial fraud detection was described by Ngai et al. [19]. The survey carried out by Ahmed et al. [20] provides an overview of anomaly detection methods, specifically clustering algorithms, in the financial domain. In addition, it gives a review of anomaly detection methods application on big data in financial markets. Subsequently, Ahmed et al. [21] defined assumptions on how to detect anomalies and summarised works applying partition-based and hierarchical-based clustering algorithms. Abdallah et al. [22] proposed a survey on fraud detection systems. In addition, Gai et al. [23] proposed a very comprehensive survey on Fintech technology in general, whereas Ryman-Tubb et al. [24] provided a survey on credit card fraud detection. Then, West and Bhattacharya [25] presented survey results of applying classification algorithms to financial fraud detection. Additionally, they analysed the strengths and limitations of a classification-based approach to financial fraud detection and classified existing works in terms of performance, applied algorithms, and fraud types. A general overview about graph-based anomaly detection methods was presented by Pourhabib [26]. Long short-term memory (LSTM) is also one of most recently studied methods within the Fintech domain [27,28].

### 2.1. Credit Cards

Credit cards have become a widespread payment method on the online market. Goods and services can be bought easily, whereas a financial institution keeps track of payment history and offers protection to customers [29]. Because of these advantages, credit cards have become increasingly popular over the years. Unfortunately, the rate of cybercrime in credit card-related payments also follows this trend. One type of fraud is facilitated by the illegal possession of credit card details. In most cases, the fraudulent user is not even in possession of the credit card [30]. Thus, detecting and tracking frauds represents a challenging task for several reasons. First, the behaviour of legitimate and fraudulent users is indeterministic over time. This means that the analysed credit card data are usually heavily imbalanced [31]. In such a way, frauds lack a consistent pattern, which makes them hard to detect. Another problem is that just a relatively small percentage of credit card transactions is not genuine. The most intuitive approach would be to maintain and track user profiles in order to monitor unusual behaviour. However, this represents a further problem due to the vast number of existing credit card users [7].

For this reason, intelligent ML algorithms are applied in order to address this challenge. These methods are used in order to identify suspicious transactions from the vast number of analysed payments. Unfortunately, the majority of alerted payments cannot be verified due to time and cost constraints [16]. Other technical problems include misclassification errors, e.g., labelling a fraudulent transaction as genuine. A typical issue represents overlapped data, i.e., generation of false positives and negatives [29]. This issue, as well as the lack of standardised metrics, is addressed further in Section 4. The following papers address credit card frauds in an ML manner.

Singh and Jain, ref. [32] enumerated and analysed work that applies adaptive machine learning (AML) techniques for credit card fraud detection. They analysed their performance with regard to sensitivity, specificity, and accuracy. Lucas and Jurgovsky [33] provided a survey on ML methods for fraud detection in credit cards. They put the emphasis on the challenges for detecting frauds with regard to unbalanced datasets. Since the behaviour of the credit card holder can change over time, this leads to the mentioned dataset shift. This fact complicates the application of ML so methods are elaborated that capture sequential properties of transactions. Other research summarises and discusses the application of AI techniques to fraud detection in credit cards [29,31,34].

A paper that discusses calibrated probabilities is described in the work of Bahnsen et al. [35]. In this case, two different methods for calibrating probabilities are evaluated and analysed in the context of credit card fraud detection. The goal of the approach is to find a model that minimises fraud-caused impact. After calibrating probabilities, the authors applied the Bayes minimum risk classifier to reduce the risk of such impacts. Finally, they claimed that this method outperforms traditional ML techniques, which often rely on raw probabilities and fixed thresholds.

Dal Pozzolo et al. [36] analysed how undersampling affects the posterior probability of a ML model. They applied a technique which is able to produce well-calibrated classifiers that play an important role for fraud detection. The same group of authors published several other papers within this domain and tested applications of different ML and DL techniques for fraud credit-card fraud detection [15,16,37,38,39]. Puh and Brkić [30] addressed the problem of credit card fraud detection in e-commerce. They compared the efficiency of several ML methods on the mentioned real-world dataset. In addition to common ML algorithms, they applied the SMOTE sampling method for anomaly detection. The authors applied static and incremental learning in order to derive a model. Afterwards, the methods are evaluated by applying ROC curve (AUC) and average precision (AP) measures. Subsequently, they evaluated the used algorithms with regard to precision and recall. They concluded that SVM achieves the lowest results, while RF and LR result in a draw.

Chalapathy and Chawla [7] discussed Deep Learning (DL) for inspection of credit card transactions. In fact, the lack of consistent patterns represents the biggest challenge for this type of fraud. For this sake, techniques from Deep Anomaly Detection (DAD) are used to track the user’s profiles and behaviour for deviations. Anomalous behaviour in data flows is investigated by applying Group anomaly detection (GAD). GAD puts an emphasis on irregular group distributions by investigating collections of individual data points. Another DL approach was described in the work of Roy et al. [40], which provides a guide to sensitivity analysis of model parameters. Therefore, it puts focus on the performance in fraud detection and cost reduction.

Pumsirirat and Yan [41] investigated credit card frauds by applying two DL techniques, namely the Autoencoder and Boltzmann machine. Therefore, they used the H${}_{2}$O platform [42] and the Keras API [43]. AUC is used to determine the success rate of the learnt model on multiple fraudulent datasets. Finally, they confirmed that DL methods can be successfully applied in order to accurately detect frauds.

Bhattacharyyaa et al. [44] proposed a comparison of results of applying SVM, random forest, and logistic regressions to a credit card fraud detection. Behera and Panigrahi [45] proposed a method for utilising the fuzzy c-means clustering algorithm and neural network algorithm to detect credit card frauds. On the other hand, Sahin and Duman [46] utilised artificial neural network and logistic regressions.

Carminati et al. [47] described a ML-based approach for analysing the reasons behind frauds in credit cards. The introduced banking support system investigates transactions in search for anomalies. It correlates suspicious features that separate normal behaviour from anomalous ones. For this sake, it relies on a semi-supervised and unsupervised anomaly detection approach.

Bahnsen et al. [48] proposed a framework that relies on a decision tree learning method that relies on cost-based measures. The authors claimed that their method outperforms other cost-sensitive methods with regard to financial savings.

Dai et al. [49] fused multiple existing detection models in the form of an online detection system. The resulting hybrid framework combines features from common fraud detection systems. Subsequently, the framework is implemented with Big Data technologies in a structure with four layers. The authors run their system on a simulated transaction dataset with 20 transaction attributes, including a transaction frequency. Finally, they discussed the performance of their real-time framework and provide further ideas for existing challenges.

Xuan et al. [50] analysed two types of random forest algorithms for training of different behavioural features. The methods are applied on normal and fraudulent transactions from a Business-to-Consumer (B2C) dataset. Subsequently, both methods are compared with regard to the obtained performance.

Ryman-Tubb et al. [24] addressed fraud detection in credit card transactions from a different, i.e., non-technological, perspective. They pointed to the emergence and impact of fraudulent patterns in criminal activities from a societal aspect. In addition, they claimed that such patterns lead to illegitimately acquired funds, which in turn can be tracked to serious and violent crime. In such way, they cause an unpredictable impact on society and industry. Therefore, the authors provided research guidelines on how to address fraud detection according to different technological levels.

Zhang et al. [51] proposed a feature engineering framework based on a homogeneity-oriented behaviour analysis (HOBA) and tested several different deep learning methods such as CNNs, RNNs and DPNs for the detection of fraudulent transactions. Their results show that their initial feature engineering with HOBA significantly improve the results. Lucas et al. [52] examined model credit card transactions from three different perspectives using Hidden Markov Models (HMMs). The presented approach can be considered as an automated feature engineering for modelling temporal correlations. The proposed automated feature engineering strategy can be used to enable a non sequential classifier to use sequential information.

### 2.2. Financial Transactions

Besides credit card cybercrime, other types of frauds exist in the virtual domain of Fintech. These include, for example, money laundering and online auction fraud. The latter encompasses, among others, fake transactions, fraudulent refunds and loans, non-payment and unauthorised purchases [53]. Internal acts of committed cybercrime within companies are known as occupational fraud. Usually, such acts are conditioned by illegitimate acquisition of confidential user data. The challenge in detecting such frauds lies in the fact that Fintech activities are committed over an interactive network of trade. In such way, a fraud can be correlated to any user, item or time [54]. This is especially problematic in free trade zones, i.e., countries with loosely or unregulated markets. To supplement the fraud detection process, policies such as anti-money laundering (AML) have to be implemented and enforced at state level [55]. The following papers tackle this problem by applying ML-based approaches to financial datasets.

Magomedov et al. [56] proposed an anomaly detection method in fraud management based on ML and graph databases. A paper with the same motivation, which focuses on money laundering, was presented by Huang et al. [57]. They introduced a detection framework, called CoDetect, that analyses a network, i.e., its entities and transactions, and subsequently detects frauds and feature patterns. CoDetect applies a Graph mining approach for different real-world fraud scenarios. Another general discussion about the use of ML for fraud detection in financial transactions was set out by Amarasinghe et al. [58]. La and Kim [59] proposed a comprehensive framework for managing Fintech transactions which utilises machine learning-based intelligence in deriving anomaly detection models and adaptive Fintech security provision.

Le Khac and Kechadi [55] applied *k*-means algorithms to detect money laundering whereas Chang and Chang [60] used the same method to detect online auction frauds. In addition, Chang and Chang [53] proposed a method for early fraud detection in online auctions. They reduced attributes used for generating learned models through principal analysis and utilised the last 20% of the transaction histories in building the models to maximise detection rates while minimising efforts. Some authors use hybrid approaches to maximise the fraud detection performance. On the other hand, Glancy and Yadav [61] and Torgo and Lopes [62] utilised hierarchical clustering for anomaly detection in financial transactions. Yaram [63] proposed document clustering and classification algorithms for identifying frauds in insurance claims.

Xu et al. [64] discussed a relatively novel type of financial fraud, namely in peer-to-peer (P2P) money lending. P2P lending takes place on an online marketplace, where loans are acquired without financial institutions in-between. This discussion is important because P2P lending has not attracted much research interest on fraud detection. First, the authors elaborated existing detection methods, including ML-based, in this context. Afterwards, they provided possible research directions with regard to fraud detection in such environments.

Leite et al. [65] conducted a comprehensive study of existing visual fraud detection approaches. The work puts a focus on visualisation techniques such as line plots, node-link diagrams, scatter plots, etc. In addition, the authors provided a comparative assessment of each approach. Finally, they concluded that most of the elaborate approaches do not integrate automatic methods for fraud detection. Other approaches that discuss visual analytics to fraud detection in real-time [54,66].

Wedge et al. [67] proposed an approach for automated feature engineering designed to reduce the number of false positives. The authors stated that accessible information about cards and customers can increase the size of potential features set drastically. The manual extraction of features is time consuming, and there might exist a need to repeat the procedure several times, i.e., every time a new bank is added in a dataset. To address this issue, the authors proposed an automated method for feature engineering, namely Deep Feature Synthesis (DFS). The results show a decrease of false positives by 54% on a previously unseen dataset consisting of 1952 million transactions. Long et al. [68] also considered feature engineering using Deep Learning. The authors proposed an end-to-end model for the feature extraction from financial time series samples and price movement prediction, using convolutional and recurrent neurons—a multi-filter neural network. Baesens et al. [69] stated that data engineering is crucial to improve the performance of the most of machine learning models. In their paper, a data engineering process consisting of several feature and instance engineering steps is proposed and demonstrated on a payment transactions dataset from a large European bank.

### 2.3. Blockchain

In the world of Fintech, the most novel payment technology comes in the form of blockchain which provides the technological backbone for the bitcoin cryptocurrency. This digital currency is exchanged over a decentralised P2P network so no central authority is needed to intermediate the transaction [70]. Blockchain relies on encryption in order to ensure security for interacting peers. Also, it establishes a distributed consensus in the network that ensures that rules are applied to incoming data blocks. Thus, transactions over blockchain are often considered to offer a higher degree of security [71]. Unfortunately, this is not always the case. Until now, several successful attacks on blockchain have been recorded within the Fintech domain.

Attacks such as the 51% attack [72], Goldfinger attack [73] or feather-forking [74] prove that blockchain is not resilient to fraudulent exploitation. Relatively few approaches exist that target anomalies in the blockchain domain. Due to its complexity, however, many intelligent techniques are applied to address these challenges. A broad overview about existing threats and ML solutions in the bitcoin environment was given by Rahouti et al. [75]. In the following, we provide an overview about the application of several ML methods for the detection of cybercrime in the bitcoin ecosystem.

Pham and Lee [76] applied ML techniques to detect suspicious users and transactions in financial networks. Specifically, the authors applied *k*-means clustering, Mahalanobis distance and unsupervised Support Vector Machine (SVM). The approach is demonstrated on two case studies from a bitcoin framework with promising initial results. Furthermore, Ostapowicz and Żbikowski [77] used supervised learning techniques to detect fraudulent accounts on blockchains. This work compares models such as Random Forests, SVM and XGBoost classifiers to identify suspicious accounts. Similarly, Monamo et al. [78] used a trimmed *k*-means unsupervised learning mechanism for anomaly detection in a bitcoin network. This method is capable of simultaneous object clustering, thus achieving positive results for fraud detection in such transactions.

Bartoletti et al. [79] addressed a classic fraud, the Ponzi scheme, in the context of blockchain. Supervised learning approaches are applied to automatically identify Ponzi schemes. First, a collection of bitcoin addresses is gathered. Then, datasets are constructed from these addresses by extracting important features. The detection model for the bitcoin Ponzi scheme is defined as a binary classification problem. Afterwards, applied ML algorithms are evaluated with regard to their effectiveness in anomaly detection. Another work that discusses this type of fraud was presented by Chen et al. [80]. Here, an ML method is applied to detect such schemes on a famous blockchain platform. First, source code from real-world samples is used to obtain the ground truth. Then, an RM model is built that is used to identify anomalous schemes. In such a way, early warnings of scams can be generated in order to detect encountered frauds in time.

Podgorelec et al. [81] focussed on frauds in blockchain transactions by introducing ML-based signing and information monitoring. To this end, they applied Isolation Forest, an unsupervised anomaly detection method. The technique simplifies the digital signing process by automatically executing the process. An anomaly detection model is created, which is used to evaluate transactions for anomalies. Subsequently, this personalised anomaly detection process tracks transactions for individual user profiles.

Meng et al. [82] discussed security-related issues and intrusion detection in blockchain architectures.

## 3. ML Methods for Financial Fraud Data Classification

### 3.1. Machine Learning Tools and Algorithms

One of the greatest challenges for fraud detection is the fact that it requires real-time processing [7]. In general, the accuracy of manual fraud detection techniques is relatively low. This task is quite demanding with regard to time and resources to identify common fraud patterns. Another challenge represents the fact that profiles of common and fraudulent behaviours are subject to constant change. In addition, existing information about frauds is often skewed and cannot be relied on. Various ML methods have been proposed in literature. They can be categorised as general ML methods, ensemble approaches, graph-based, outlier detection and deep learning methods. The performance of automated fraud detection is determined by the sampling approach, the selection of variables and anomaly detection techniques [31]. Another problem in ML-based methods represents the fact that suspicious transactions are not immediately reported. In such a way, frauds remain unaffected until reported by customers [16]. In the following subsections, an overview of anomaly detection and ensemble approaches is provided that addresses these issues.

#### 3.1.1. Outlier Detection Methods

This subsection provides a short description of several anomaly detection methods that are used for later experiments. In addition, an overview is given on unsupervised outlier detection methods and novelty detection methods as part of a one-class classification (OCC). In the latter, the training data contains just normal data so an outlier check needs to be made for every new observation.

##### Local Outlier Factor

The Local Outlier Factor (LOF) method was initially proposed by Breunig et al. [83] for moderately high dimensional datasets. To reflect the degree of abnormality for the observation of an object locally (and not just globally on the whole dataset), a local outlier score (LOF) is calculated. The approach is local in the sense that only a restricted neighbourhood is taken into account for the LOF score of each object. This is achieved by considering the *k*-nearest neighbours, a comparison of the local density of an object and the local density of its neighbour’s objects. In the case that an object has a significantly lower density than its neighbours, it is considered to be an outlier.

One of the very important parameters in this method is the value *k*, which needs to be set correctly. A too high value for *k* will detect just global outliers, whereas a low *k* results in the detection of outliers in small regions, which increases the overall false positive rate. In addition, a minor similarity exists between LOF and density-based clustering methods, such as OPTICS [84] or DBSCAN [85].

##### Isolation Forest

Another suitable method for outlier and novelty detection is Isolation Forest, which was proposed by Liu et al. [86]. This method is also suitable for high dimensional datasets. Isolation Forest does not—as many other methods—construct a profile or normal behaviour. It isolates anomalies explicitly by relying on the fact that anomalies represent a minority in the dataset and that they have attribute values very different from normal ones. The isolation is performed in a tree structure, where anomalies that are closer to the root of the tree are isolated due to higher susceptibility than normal points. For this reason, the latter are isolated at the deeper end of the tree. After building an ensemble of trees for a given dataset, anomalies are recognised by having a short average path length.

##### Elliptic Envelope

Another suitable method for outlier detection represents Elliptic Envelope [87]. Generally, this method is applied for Gaussian distributed regular data which in addition must not be high-dimensional.

Under the assumption that the data are of Gaussian distribution, Elliptic Envelope fits an ellipse around the data with the help of robust covariance estimation. Any data point inside the ellipse is considered as inliers, whereas data points outside the ellipse are outliers. For fitting in an ellipse, a contamination parameter is used, determining the amount of data which will be inside the ellipse.

#### 3.1.2. Ensemble Approach

Ensemble methods are the most popular techniques that are used in machine learning. They combine multiple models to build an optimal predictive model that is powerful in terms of both computation and efficiency.

##### Random Forest

The Random Forest (RF) method builds a forest of individual decision trees that collectively constitute an ensemble. Each of the trees makes a prediction on the data by taking majority votes. In turn, the class with the majority vote is decided as the final prediction. Thus, individual uncorrelated models come together to perform the best prediction on the data. Random forest maintains the correlation between trees as minimal as possible by employing the technique of “bagging”. As decision trees are sensitive to training data, Random forest builds the trees on randomly sampled data with replacement. This results in different trees with minimum correlation amongst them. Moreover, Random Forest maintains “Feature Randomness” because each tree makes its decision based on a random subset of features rather than picking every feature from the dataset. In such way, great variation in the factors is ensured on which each tree is built on. Thus, bagging and feature randomness enable the random forest model to train not only with random samples of a dataset but also with different subsets of features for prediction, whilst hyperparameter tuning of a sufficiently large parameter set prevents it from overfitting on the data.

##### Adaptive Boosting (AdaBoost)

AdaBoost serves the objective of evolving a strong classifier based on a set of weaker classifiers. The common method used with AdaBoost is the decision tree. It develops a strong classifier by with the weighted combination of the set of weak classifiers. First, the algorithm tries to fit the training data on a set of classifiers. Then, it picks the one with the least weighted classification error and updates the weights on other data points. This is done by using a normalisation factor that ensures that the sum of all the weights of the data points is equal to 1. Thus, after each iteration, the model attempts to minimise the classification error of the classifiers. This process is repeated until the training dataset is classified appropriately or no further pruning can be carried out on the training dataset. A dataset with outliers would undermine the performance of the AdaBoost on test/unseen data by degrading the ability of the algorithm to rectify the misclassification errors at each iteration. Similarly, any noise in the prediction variable would cause issues with the performance of the algorithm. Thus, outliers and noise in the training data have to be removed before applying AdaBoost.

##### Extreme Gradient Boosting (XGBoost)

XGBoost is an ensemble ML algorithm based on the concept of decision trees, similar to Random Forest and other Boosting algorithms. XGBoost achieves significant results for classification problems because it applies the principle of boosting a set of weak trees by using a gradient descent approach. Gradient Boosting generally attempts to weed out the less favourable trees with the aim of minimising errors with a gradient descent algorithm. XGBoost further improves this framework through algorithm enhancements and additional optimisations. In general, such improvements offer many advantages. Algorithm level enhancements include:

- Regularisation: This process penalises models to avoid overfitting.
- Sparsity Awareness: XGBoost deals with sparse input features by learning according to the training loss; it also handles various sparsity patterns in the dataset more efficiently.
- Weighted Quantile Sketch: XGBoost find the best splits for the dataset by employing the distributed weighted Quantile Sketch algorithm.
- Cross Validation: XGBoost does cross-validation on the dataset by default rather than using a separate mechanism to search for the exact number of iterations.

In addition to the above factors, XGBoost supports various system level optimisation techniques.

### 3.2. Pre-Processing Tools and Algorithms

Getting an initial overview of the data including the selection of suitable pre-processing methods to fulfil the assumptions to feed the data to a Machine Learning Model is crucial for obtaining meaningful results. This section focusses on suitable visualisation methods as well as methods for encoding categorical data.

#### 3.2.1. Data Statistics and Visualisation

To get an overview about the data, find issues which might need to be addressed (e.g., not number values) and to select suitable algorithms in a first step some statistic of the data, as well as a visualisation is performed. This includes checking the data types (categorical values usually need pre-processing before being fed into certain Machine Learning methods), the number of samples for the normal and the abnormal class and especially for numerical values statistical values such as mean, std, min, max and certain quartiles to get insight into the potential distribution of these features.

In the case of numerical features, univariate plots such as box plots and histograms give a quick visualisation of the statistical parameters and their potential distribution. To study the interaction between (numerical) features, multivariate plots such as scatter matrices can provide information. For a potential feature selection or reduction step, a correlation analysis between the features or dimensionality reduction methods as PCA (Principal Component Analysis) can provide insights as well as help to reduce the number of features.

Since box plots are used in this paper, a more detailed explanation is given here. Box plots are a useful state-of-the art visualisation technique for gaining insights into the distribution of features. By presenting the median values as the central line of the box with the boundaries of the first and third quartile, representing the median of the lower half of the dataset and the median of the upper half of the dataset, respectively, it shows where 50% of the data are lying. The box plot further has some whiskers, for which there are different definitions. The focus here is on the definition where the whiskers are maximal 1.5 of the inter-quartile distance in both directions. The corresponding whisker line is then at the place where the last suitable data point within this distance appears. Other data points are marked with dots as outliers.

Box plots, in particular, give an insight of the kind of the distribution (e.g., skewed or symmetric) and can indicate a normal distribution of some features. Therefore, those plots give indicators if a certain method (e.g., under the assumption that features follow a normal distribution) can be used. In particular, when using parameters as contamination factors, it can give an estimate about the potential percentage of outliers within the dataset.

#### 3.2.2. Feature Engineering—Categorical Variable Encoding

To use categorical variables for many Machine Learning algorithms, a pre-processing step to convert those values into numerical ones is necessary. There are several possible ways to convert categorical variables. Since the encoding is likely to influence the performance of the algorithms [88,89], it has to be chosen carefully. In general, there are two categories for the encoding of categorical variables:

- Nominal Encoding: There is no order between the categories (e.g., colours).
- Ordinal Encoding: There is some order (a sequential indication).

Some common categorical encoding methods are listed in Table 1. For a more extensive list of encoding techniques, one can refer to the work of other researchers [89,90,91]. It has to be emphasised that the cardinality of variables has to be taken into account, since most of them are not suitable for high cardinality due to the so-called curse of dimensionality.

In the following text, two techniques for encoding unbalanced datasets are described in more detail: the Leave One Out Encoding and the Weight of Evidence algorithm.

##### Leave One Out Algorithm

The Leave One Out algorithm calculates the mean of the target variable for all records in a given category except the current record. The regularisation factor *R* and the randomness factor ϵ are used to prevent over-fitting. One can refer to the work of Hancock et al. [89] for more information.

(1) $$\begin{array}{c} \nu_{i}=\left(\sum_{j!=i}\frac{t_{j}}{n-1+R}\right)x\left(1+\varepsilon_{i}\right) \end{array}$$

with

$$\begin{array}{cc} \nu_{i} & \ldots \mathrm{encoded}\;\mathrm{value}\;\mathrm{for}\;\mathrm{the}\;i\mathrm{th}\;\mathrm{record}\\ t_{j} & \ldots \mathrm{target}\;\mathrm{variable}\;\mathrm{for}\;\mathrm{the}\;j\mathrm{th}\;\mathrm{record}\\ n & \ldots \mathrm{number}\;\mathrm{of}\;\mathrm{records}\;\mathrm{with}\;\mathrm{the}\;\mathrm{same}\;\mathrm{categorical}\;\mathrm{variable}\;\mathrm{value}\\ R & \ldots \mathrm{regularisation}\;\mathrm{factor}\\ \varepsilon_{i} & \ldots \mathrm{zero}\;\mathrm{mean}\;\mathrm{random}\;\mathrm{variable}\;\mathrm{with}\;\mathrm{normal}\;\mathrm{distribution}\;N(0,s) \end{array}$$

The algorithm in Equation (1) only works in supervised manner. For the validation dataset or the test dataset a slightly different encoding is used, namely

(2) $$\nu_{i}=\frac{\sum_{j}^{N}t_{j}}{n+R}$$

##### Weight of Evidence Algorithm

This method separates the positive and negative class, considering positive as “good” and negative as “bad”. The Weight of evidence (WOE) gives a measure how a certain value supports or under determines a hypothesis. It is a univariate approach and therefore does not consider potential correlations between independent variables.

(3) $$\nu_{i}=\log\left(\frac{\frac{p_{i}}{p}}{\frac{n_{i}}{n}}\right)$$

with

$$\begin{array}{cc} p_{i} & \ldots \;\mathrm{number}\;\mathrm{of}\;\mathrm{records}\;\mathrm{with}\;\mathrm{positive}\;\mathrm{class}\;\mathrm{value}\;\mathrm{for}\;\mathrm{the}\;\mathrm{considered}\;\mathrm{categorical}\;\mathrm{attribute}\;\mathrm{value}\\ n_{i} & \ldots \;\mathrm{number}\;\mathrm{of}\;\mathrm{records}\;\mathrm{with}\;\mathrm{negative}\;\mathrm{class}\;\mathrm{value}\;\mathrm{for}\;\mathrm{the}\;\mathrm{considered}\;\mathrm{categorical}\;\mathrm{attribute}\;\mathrm{value}\\ p & \ldots \;\mathrm{total}\;\mathrm{number}\;\mathrm{of}\;\mathrm{records}\;\mathrm{with}\;\mathrm{positive}\;\mathrm{class}\;\mathrm{value}\\ n & \ldots \;\mathrm{total}\;\mathrm{number}\;\mathrm{of}\;\mathrm{records}\;\mathrm{with}\;\mathrm{negative}\;\mathrm{class}\;\mathrm{value} \end{array}$$

#### 3.2.3. Feature Selection—Information Value (IV)

The Weight of Evidence Algorithm, described in previous section, is related to the Information Value. The Information Value is a very useful technique giving a rank of importance of variables for a predictive model [92]. It is calculated with

(4) $$IV=\sum \left(\%\mathrm{of}\;\mathrm{non-events}-\%\mathrm{of}\;\mathrm{events}\right)*WOE$$

In credit scoring, the values of the IV statistics can be interpreted based on Table 2 according to Siddiqi [92] and Brotherton et al. [93].

It has to be emphasised that the predictiveness of the Information Value in Table 2 has been developed for credit scoring. The goal of the features there is to indicate if a certain set is a good or bad candidate for a credit. This is a different problem formulation than the automated fraud detection based on different types of transactions, in which case, all features are automatically generated by the system. In consideration of the above, these IV rules will be used to choose the minimal and most optimal feature subset for detecting frauds in transactions.

### 3.3. Reliability of Anomaly Detection Algorithms

Machine learning (ML) algorithms differ in their ability of explaining a specific output. Such an output can be the prediction of a class membership for a certain instance. An explanation seeks to answer the question which input features contributed to what extent towards or against the assignment of the instance to a given class. The predictions of algorithms such as Support Vector Machines (SVMs) or Decision Forests are suitable for giving such an explanation as the algorithms themselves are comprehensible for a human evaluator. Other algorithms such as Deep Neural Networks (DNNs) typically remain black boxes as they lack this kind of explainability. The complex inner structure and vast number of computations and parameters makes it hard to answer the question of what exactly caused the neural network to output a prediction for a specific instance.

In the context of detecting fraudulent transactions within a financial network, the information as to which input features contributed to what extent towards or against a classification as fraud can help the human operator to assess the reliability of the output. Therefore, it is easier for the operator to see whether the tagging of a transaction as fraudulent is based on meaningful patterns in the input features or not. A transaction which was identified as fraudulent because of the features “time” and “amount of money” is possibly more in accordance with the expert knowledge and experience of the operator than a tagging as fraudulent because of the features “currency” and “IBAN”. This helps the operator to quickly identify false positives and to lower the rate of false alarms.

In ML, different approaches exist that explain the output of a trained model. The method LIME (Local interpretable model-agnostic explanations) proposed by Ribeiro et al. [94] approximates a classification boundary of a black-box model around a specific prediction, whereas SHAP (SHapley Additive exPlanations) proposed by Lundberg et al. [95] assigns importance values to each feature according to its contribution against or towards a specific prediction. Recent work especially in the field of financial transactions includes a method called MANE (Model-Agnostic Non-linear Explanations for Deep Learning Model) proposed by Tian et al. [96], which follows a multi-level approach including historical transaction data. For reliability analysis in this paper, the method of Layer-wise Relevance Propagation proposed by Lapuschkin et al. [97,98,99] will be deployed.

#### 3.3.1. Reliability Analysis with Layer-wise Relevance Propagation (LRP)

To achieve the above-mentioned goal, the method Layer-wise Relevance Propagation is deployed for the use case of detecting fraudulent transactions with neural networks. Lapuschkin et al. [97,98,99] provided more information on the theory of Layer-wise Relevance Propagation. On the other hand, Alber et al. [100] and Lapuschkin et al. [101] elaborated on the implementation details of the algorithm.

#### 3.3.2. Methodology

To deploy the reliability analysis with Layer-Wise Relevance propagation, a fully connected neural network with an architecture as described in Table 3 is used.

The number of neurons of the input layer is equal to the number of selected features. The total number of trainable parameters of the neural network is 112,602. These parameters are initialised with a Glorot uniform initialiser [102] which draws samples from a uniform distribution within [−*r*, *r*], where

(5) $$r=\sqrt{\frac{6}{n_{\mathrm{input}}+n_{\mathrm{output}}}}$$

with $n_{\mathrm{input}}$ the number of input units and $n_{\mathrm{output}}$ the number of output units. As an optimiser, the Adam algorithm [103] is used with a learning rate of 0.001. The neural network is trained for 100 epochs. The input to the LRP-algorithm are the weight matrices of connections between subsequent layers and the activation functions for each layer. The output are Relevance scores for each transaction of the test set for each feature describing the contribution towards (positive score) or against (negative score) the classification. The sum of absolute Relevance scores is normalised to 1.

The analysis of the Relevance scores aims to establish whether it is possible for a human operator to gain indications for a misclassification by looking at the Relevance scores of the considered transaction. The Relevance scores of the test are therefore analysed with respect to the distribution of ratios $R_{i}^{sign}$ between the number of negative and positive Relevance values

(6) $$R_{i}^{sign}=\frac{\mathrm{number}\;\mathrm{of}\;\mathrm{negative}\;\mathrm{Relevance}\;\mathrm{scores}\;\mathrm{for}\;\mathrm{instance}\;i}{\mathrm{number}\;\mathrm{of}\;\mathrm{positive}\;\mathrm{Relevance}\;\mathrm{scores}\;\mathrm{for}\;\mathrm{instance}\;i}.$$

If the number of positive Relevance values is zero, the ratio is set equal to the number of features in order to avoid undefined behaviour. The distributions of the sum of positive Relevance scores are analysed as well.

## 4. Evaluation Methodology

### 4.1. Experiment Workflow

ML-based fraud detection in the fintech domain in general consists of several common blocks, which are also part of the work presented in this paper:

- Case studies selection: Case studies selection is based on availability of publicly available datasets; due to a lack of availability of real data within this domain, synthetic data creation by domain experts is often used as a way to overcome this issue. Therefore, this section includes an overview of publicly available datasets in fintech domain.
- Data collection: This step presents gathering information regarding a specific case study; available datasets and specific scenarios are identified, including data collection/creation procedures and fraud scenarios. This step includes identification of fraud indicators that can be used as features within dataset.
- Data statistics and visualisation: Various statistics and visualisation techniques are performed on data, in order to understand data and pre-process it for optimal use.
- Feature engineering and selection: This includes feature investigation and selection; categorical data encoding, as a necessary pre-processing step for some ML methods; and selection of the most optimal feature subset based on feature influence on detectability.
- Algorithm selection: Several ML based algorithms are tested to find those most suitable to detect financial transaction frauds. The experiments presented in this paper included outlier detection approaches (Local Outlier Factor, Isolation Forest and Elliptic Envelope) and ensemble approaches (Random Forest, Adaptive Boosting and Extreme Gradient Boosting).
- Evaluation: The most commonly used evaluation metrics are selected, namely true positive rate (TPR) or sensitivity, true negative rate (TNR) or specificity and Receiver Operating Characteristic (ROC) curve for graphical presentation. Although there are no “perfect” metrics that reflect all aspects of fraud detection problem, the selected ones reflect the number of genuinely and falsely classified frauds, and provide to the scientific community a common method to compare results and build on these findings.

It should be noted that the common problem in ML applications in Fintech domain is the class imbalance in datasets. This problem can seriously affect the performance of trained models. This issue is addressed in work proposed in this paper by the careful selection of algorithms used, with the focus on one-class outlier detection methods. The training of these algorithms is based only on benign data—the goal is to learn expected characteristics of data, and based on that detect anomalies. The class imbalance is also taken into account in the evaluation by selecting the optimal metrics, e.g. TPR, TNR and ROC curves, because some common metrics such as accuracy are very misleading for highly imbalanced datasets.

### 4.2. Publicly Available Datasets

The one of the biggest challenges in Fintech research is the lack of publicly available datasets. This is mostly due to the privacy issues considering that these datasets can contain sensitive and personal data. Publicly available datasets that are not outdated, to the best of our knowledge, are listed below.

#### 4.2.1. Credit Card Fraud Detection

The dataset for the credit card fraud detection (*CreditCard* dataset in the following text) contains transactions made by credit cards of European cardholders in September 2013. This dataset contains 492 frauds out of 284,807 transactions within two days, which makes the dataset highly unbalanced, as the positive class account of all transactions is 0.172%. Dataset details are presented in Table 4.

Each transaction consists of 31 numerical features, of which 28 are PCA-transformed ones because of confidentiality issues (V1–V28) while the others are Time (indicates the elapsed time between the first transaction in the dataset and the others, respectively), Amount (Transaction Amount) and Class (represents the output variable denoting whether fraud or not) [9].

#### 4.2.2. Synthetic Financial Datasets for Fraud Detection

In this section, a synthetic dataset generated using a simulator called PaySim (*PaySim* dataset in the following text) is presented. PaySim uses aggregated data from the private dataset to generate a synthetic dataset that resembles the normal operation of transactions and injects malicious behaviour to later evaluate the performance of fraud detection methods. This is achieved by simulating mobile money transactions based on a sample of real transactions of an African country. The original data were provided by a multinational mobile financial service provider [104]. In this specific dataset, the fraudulent behaviour of the agents is intended to profit by taking control or customers’ accounts and try to empty the funds by transferring to another account and then cashing out of the system. Dataset details are presented in Table 5.

The dataset covers a range of financial transactions occurring over a 30-days period containing 6,362,620 transactions, of which 8213 transactions are fraudulent. Each transaction consists of 11 features as follows: (1) step maps a unit of time in the real world, in this case one step is 1 h of time with 744 total steps (30 days simulation); (2) type indicating the type/mode of the transaction; (3) amount denoting the amount of the transaction; (4) nameOrig representing the source initiating the transaction; (5) oldbalanceOrg containing the initial balance of the source before the transaction; (6) newbalanceOrig containing the new balance of the source after the transaction; (7) nameDest represents the recipient of the transaction; (8) oldbalanceDest contains the initial balance of the recipient before the transaction; (9) newbalanceDest contains the new balance of the recipient after the transaction; (10) isFraud denotes the output variable; and (11) isFlaggedFraud indicates a flag of an illegal attempt in this dataset (transfer more than 200,000).

#### 4.2.3. Synthetic Data from a Financial Payment System

The dataset presented in this subsection is created using BankSim, an agent-based simulator of bank payments based on a sample of aggregated transactional data provided by a bank in Spain. The goal of the framework is to generate synthetic data that can be used for fraud detection research. This dataset combines normal payments with known fraud signatures and does not contain any personal information or any other disclosure of transactions. This dataset is referred as *BankSim* dataset in the following text. The dataset details are presented in Table 6.

Simulated attacks have the aim to steal an average of three credit cards and performed about two fraudulent transactions per day. As an outcome, they produced a total of 594,643 records, where 587,443 are normal payments and 7200 are fraudulent transactions, simulated over a six-month period [13].

Each transaction consists of nine features as follows: (1) category indicating the category with respect to which the transaction has been made; (2) amount denoting the amount of the transaction; (3) customer representing the source initiating the transaction along with the information regarding the source such as (4) age; (5) gender; (6) zipcodeOri containing the postcode of the source; (7) merchant representing the recipient of the transaction; (8) zipMerchant containing the post code of the recipient; and (9) Fraud denoting the output variable indicating whether the transaction is legitimate or fraudulent.

#### 4.2.4. Bank Transaction Data

Bank transaction data are an analytical tool with the aim detecting fraud transactions and money laundering. The developers wanted to build a tool which can extract bank names using the IFSC codes; get the same number transactions through the system with a Debit as well as Credit effect on same date in two different accounts and matching narration; and would categorise similar transaction on the basis of narrations. The dataset details are presented in Table 7.

The project is available on GitHub, and has the following features available [10,105]: (1) Account No. represents the account number involved in transaction; (2) Date is the date of transaction; (3) Transaction Details are the transaction narrations in bank statements; (4) Cheque No. indicates the cheque number; (5) Value Date is the date of completion of transaction; (6) Withdrawal Amount indicates the amount withdrawn; (7) Deposit Amount indicates the amount deposited; and (8) Balance Amount is the current balance of account.

## 5. Case Studies for ML-Based Fraud Detection

Case studies for testing of ML-based fraud detection are selected according to availability of fully labelled datasets, in order to perform repeatable tests that can be easily checked and contribute to our results reliability.

### 5.1. CS#1: Credit Card Fraud Detection—CreditCard Dataset

#### 5.1.1. Feature Engineering and Selection

##### Feature Engineering and Dataset Visualisation

As previously explained, *CreditCard* dataset contains 31 numerical features, representing PCA components of original features: Time, V1, V2, V3, V4, V5, V6, V7, V8, V9, V10, V11, V12, V13, V14, V15, V16, V17, V18, V19, V20, V21, V22, V23, V24, V25, V26, V27, V28, Amount, Class.

Dataset entry (credit card transaction) class is annotated with feature Class, where 0 presents “normal” transactions and 1 presents “fraudulent” transactions. Dataset contamination is 0.172%, making the dataset highly unbalanced.

The dataset spans over two days, with the feature Time presenting the seconds elapsed between each transaction and the first transaction in the dataset. As such, it does not reflect directly enough useful information, such as time in the day or cyclic nature of time (for example, the fact that 1 h comes directly after 24 h). This fact leaves open space for additional feature engineering and contributions to existing dataset.

Thus, the original feature list is extended with three features engineered and encoded from the Time feature. The first is one is directly encoded feature, tot_seconds, presenting the number of seconds from the referent time point (first transaction) in 24 h time cycle, where feature range is 0–86,399 (86,399 = 24×60×60−1).

This feature still does not reflect cyclic nature of time and the value that comes after 86,399 is 0. To solve this, two new features are created deriving a sine transform and cosine transform of the tot_seconds feature—sin_tot_seconds and cos_tot_seconds. The sine and cosine functions are called after normalising tot_seconds within 0–2π, which corresponds to one cycle, as follows

(7) $$\sin\_\mathrm{tot}\_\mathrm{seconds}=sin(\frac{2\pi \times \mathrm{tot}\_\mathrm{seconds}}{24\times 60\times 60})$$

(8) $$\cos\_\mathrm{tot}\_\mathrm{seconds}=cos(\frac{2\pi \times \mathrm{tot}\_\mathrm{seconds}}{24\times 60\times 60}).$$

This transformation can be intuitively presented as transformation of time in 2D space as a 24-h clock.

**Figure 1:**
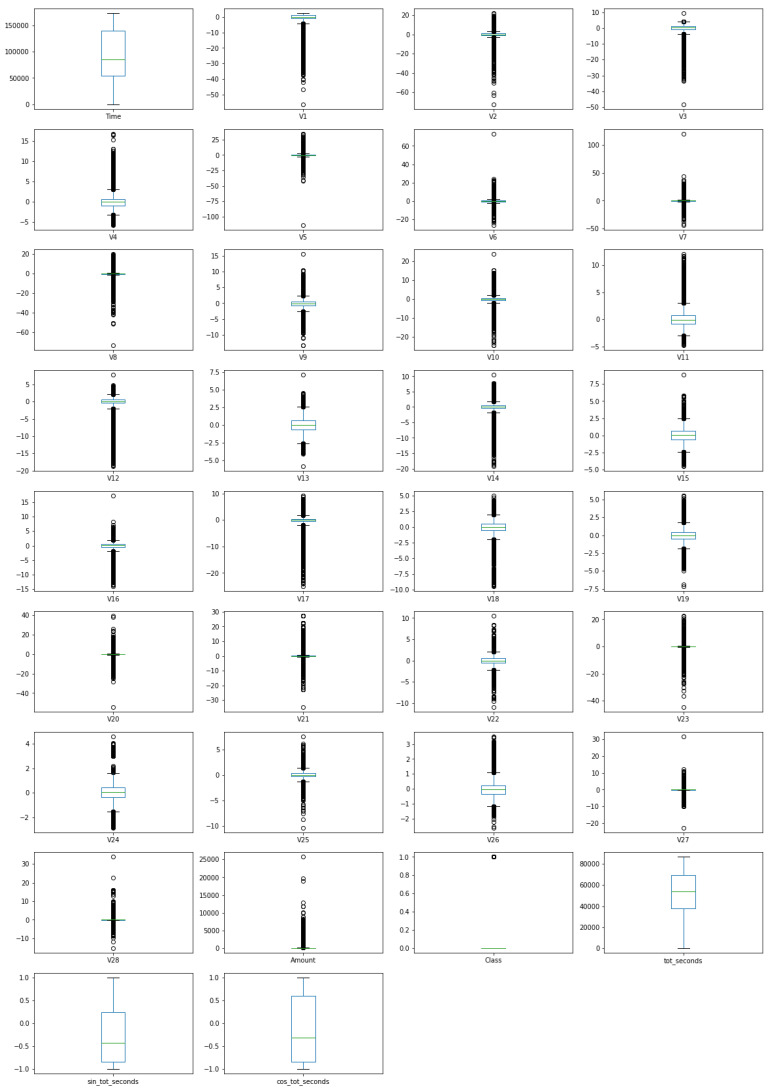
Extended *CreditCard* dataset box plots.

The following experiments were conducted on the extended *CreditCard* dataset, consisting of 284,807 entries and 34 features. Box plots of features in extended dataset are presented in Figure 1. The box plots show that the number of outliers for nearly all features (except time, sin_tot_seconds, cos_tot_seconds and tot_seconds) are really high. This indicates that a contamination factor used in the later methods should not be too low.

The conclusion that some of the features (e.g., V13 and V9) are symmetrically distributed indicates that methods such as Elliptic Envelope, that requires Gaussian distribution in data, can potentially be applied on this dataset.

Additionally, box plots indicate that feature Amount is highly skewed, and as such should be further processed before feeding it to the detection tools. Thus, Box–Cox transformation of the SciPy package is applied to transform the Amount feature closer to a normal distribution.

##### Feature Analysis

Information Value (IV) explained if previous sections calculated for all features in the extended dataset in order to estimate their potential contribution to detectability.

IV of features, presented in Table 8 and Table 9, is used in the experimental part described in the following section as indicator of feature quality and reasoning for feature selection.

#### 5.1.2. Outlier Detection Approach

The results using outlier detection algorithms applied to the extended *CreditCard* dataset are given in this section. Considering the nature of transaction flows, where there is a need to detect anomalies in the runtime environment, semi-supervised detection is more suitable, and as such it is included in tests, using 70% of randomly selected data (with excluded anomalies) in the dataset for training and 30% (including anomalies) for testing. Applying outlier detection techniques on four different subsets including non-private, numerical features gives results provided in this section.

Testing subsets are selected according to features usability from the information value point of view, described previously, with the following logic:

- *S*—dataset excluding features with *Useless*, *Weak* and *Medium* predictive power
- *S_M*—dataset excluding features with *Weak* and *Useless* predictive power
- *S_M_W*—dataset excluding features with *Useless* predictive power
- *all*—dataset with all features included

Testing subsets of the given dataset are presented in Table 10.

The results applying previously described outlier (anomaly) detection methods—Local Outlier Factor (LOF), Isolation Forest (IF) and Elliptic Envelope (EE)—on the given subsets are presented in Table 11.

The presented results indicate that feature selection is of great importance for methods such as Local Outlier Factor and Elliptic Envelope, while Isolation Forest is the most resistant one out of tested outlier detection methods.

**Figure 2:**
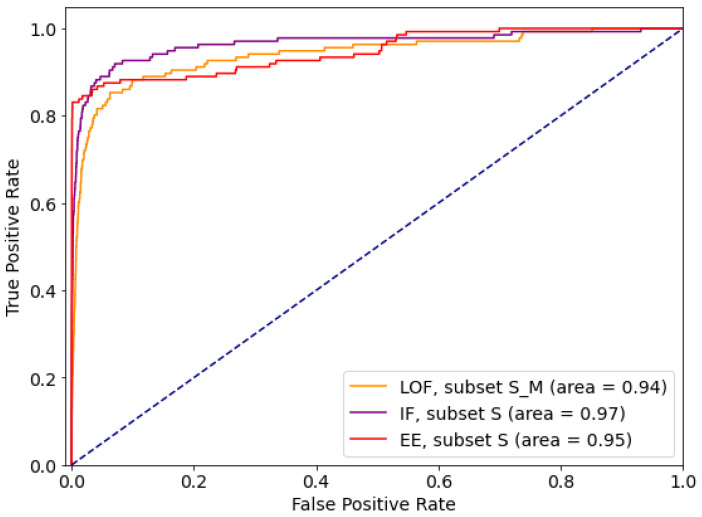
Extended *CreditCard* dataset—comparative ROC curve for outlier detection methods.

Comparative ROC curves for the best performing subsets are given in Figure 2. Comparative analysis based on *sensitivity* (*tpr*) and *specificity* (*tnr*) indicates that for the given dataset Isolation Forest performs the best out of tested outlier detection methods. At the same time, IF is resistant to feature selection, so can be considered as the outlier detection method of choice for the data with this feature list and distribution.

#### 5.1.3. Ensemble Approach

The results applying ensemble methods to the new extended *CreditCard* dataset are given in this section. Similar to the outlier detection experiments, 70% of randomly selected data are used for training and the remaining 30% for testing. Considering the nature of tested algorithms, where by an algorithm such as Random Forest actively selects the most suitable feature subset during training, these algorithms were applied on the extended dataset containing all features. Tested approaches include Random Forest, Adaptive Boosting (AdaBoost) and Extreme Gradient Boosting (XGBoost), described previously in text.

The results of applying selected ensemble methods on the given subsets are presented in Table 12.

**Figure 3:**
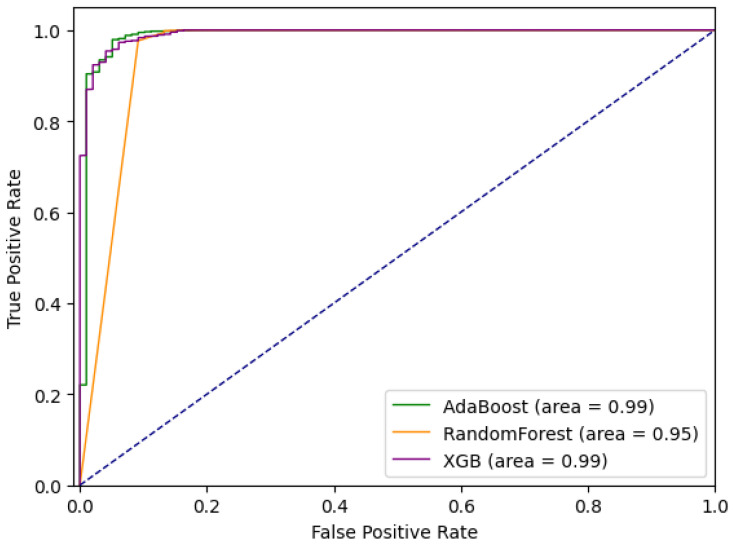
Extended *CreditCard* dataset—comparative ROC curve for ensemble methods.

Comparative ROC curves for the tested ensemble methods are given in Figure 3. Comparative analysis based on *sensitivity* (*tpr*) and *specificity* (*tnr*) indicates that for a given dataset Extreme Gradient Boosting performs the best out of tested methods. At the same time, it can be noted that sensitivity is significantly lower than specificity, and that, with the cost of missing some anomalies (approximately 20%), the number of false positives is minimal.

#### 5.1.4. Reliability Analysis

##### Training of the Neural Network

The numerical features V1–V28 and the feature Amount were scaled to a range [0, 1], whereas the feature Time was omitted.

The final dataset was split into training, validation and test sets where the test set is 20% of the whole dataset and the validation set is 10% of the training set. The total number of instances in the dataset is 284,807. This means that the number of instances in the training set is 205,061, the number of instances in the validation set is 22,785 and the number of instances in the test set is 56,961.

**Figure 4:**
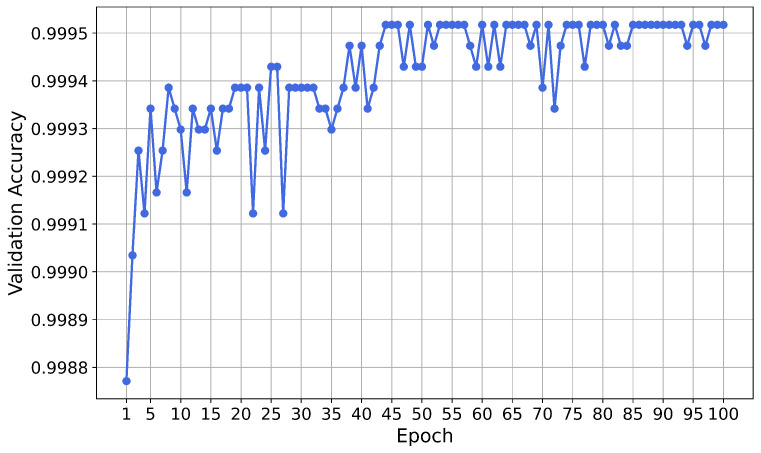
Validation accuracy of the neural network for each epoch.

The neural network was trained for 100 epochs. The validation accuracy for each epoch is shown in Figure 4. A variation of the validation accuracy of 0.0001 corresponds to an absolute variation of two predictions.

After the training phase, the performance of the neural network on the test set was evaluated. The accuracy of the test set is 0.9994, meaning that the network misclassified 34 instances out of 56,961. The ratio of fraudulent transactions which were correctly classified as fraud (true positive rate, TPR) is 0.7755 and the ratio of non-fraudulent transactions which were correctly classified as non-fraud (true negative rate, TNR) is 0.9998.

##### Results

**Figure 5:**
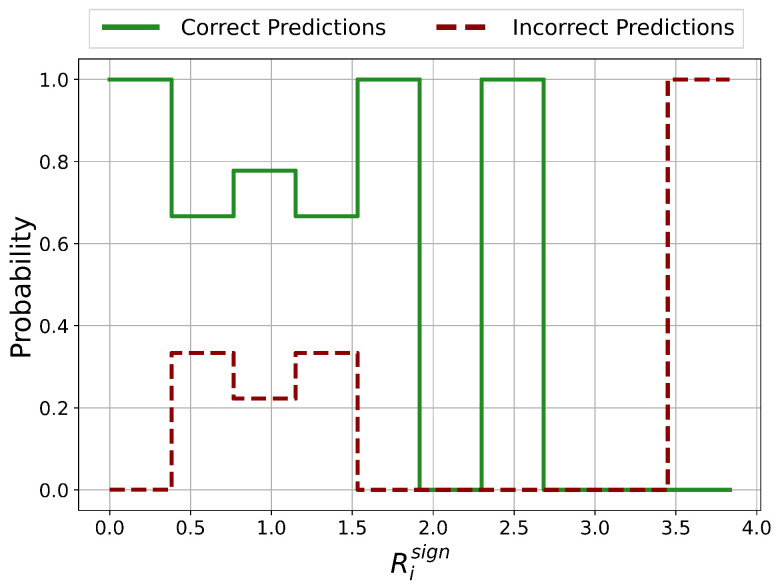
Probability distributions of ratios between the number of negative and positive Relevance scores for correct (green solid line) and incorrect (red dashed line) predictions. Potentially unreliable predictions occur in the range $0.4<R_{i}^{\mathrm{sign}}<1.5$. If $R_{i}^{\mathrm{sign}}>3.5$, the predictions are not reliable as it is certainly incorrect.

The probability distributions of ratios $R_{i}^{\mathrm{sign}}$ between the number of negative and positive Relevance scores on the test set is shown in Figure 5. If the number of positive Relevance scores is zero, $R_{i}^{\mathrm{sign}}$ is set equal to the number of features (=29).

In the approximate ranges of $R_{i}^{\mathrm{sign}}<0.4$, $1.5<R_{i}^{\mathrm{sign}}<2$ and $2.3<R_{i}^{\mathrm{sign}}<2.7$, the prediction is certainly correct. For $R_{i}^{\mathrm{sign}}>3.5$, the prediction is certainly incorrect and therefore unreliable. For $0.4<R_{i}^{\mathrm{sign}}<1.5$, it is not clear whether the prediction is correct or incorrect which represents a potentially unreliable classification.

**Figure 6:**
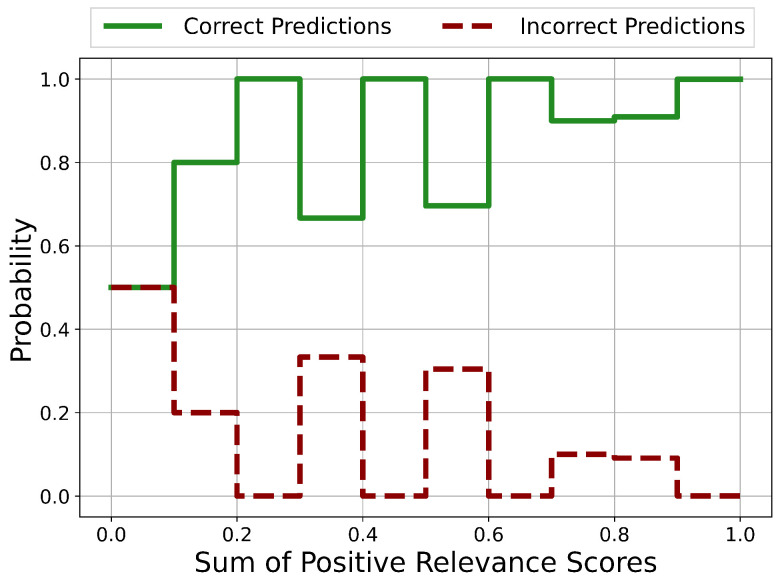
Probability distribution of the sum of positive Relevance scores in the case of correct (green solid line) and incorrect (red dashed line) predictions on the test set. Potentially unreliable predictions occur in the ranges [0.0, 0.2], [0.3, 0.4], [0.5, 0.6] and [0.7, 0.9].

The probability distributions of the sums of positive Relevance scores in the case for correct and incorrect predictions is shown in Figure 6. Potentially unreliable predictions occur in the ranges 0.0<sum<0.2, 0.3<sum<0.4, 0.5<sum<0.6 and 0.7<sum<0.9. In every other range, the prediction is certainly correct.

Except for the case $R_{i}^{\mathrm{sign}}>3.5$, there are no regions with clear unreliable and therefore incorrect predictions. Nevertheless, areas of potentially incorrect predictions and therefore with questionable reliability could be identified.

### 5.2. CS#2: Financial Transactions Fraud Detection—PaySim Dataset

#### 5.2.1. Feature Engineering and Selection

##### Feature Engineering and Dataset Visualisation

As previously explained, the *PaySim* dataset contains 11 features, representing financial transactions, namely step, type, amount, nameOrig, oldbalanceOrg, newbalanceOrig, nameDest, oldbalanceDest, newbalanceDest, isFraud, isFlaggedFraud.

Dataset entry (financial transaction) class is annotated with feature isFraud, where 0 presents “normal” transactions and 1 presents “fraudulent” transactions. Dataset contamination is 0.129%, making the dataset highly unbalanced.

The dataset spans over one month, with the feature step presenting the number of hours between each transaction and the first transaction in the dataset. As such, similar to the first case study, it does not reflect directly enough all useful information, and is a good candidate for additional feature engineering. The original feature list is extended with seven features engineered and encoded from the step feature.

There are three directly encoded features:

- hour—presenting the number of hours from the referent time point (first transaction) in 24 h time cycle, where feature range is 0–23
- day—presenting the day in the month in 30 days month cycle, where the feature range is 1–30
- weekday—presenting the day in the week in seven-day week cycle, where the feature range is 1–7

These features, and especially hour and weekday, do not reflect cyclic nature of time. To solve this, four new features are created deriving a sine transform and cosine transform of the respective features—sin_hour, cos_hour, sin_weekday and cos_weekday. The sine and cosine functions are called after normalising initial features between 0 and 2π, similar to Equations (7) and (8).

In addition to engineered time features, categorical feature type, containing five different categories, is encoded using One Hot encoding technique, adding additional five binary features to the dataset—is_type_CASH_IN, is_type_CASH_OUT, is_type_DEBIT, is_type_PAYMENT and is_type_TRANSFER.

**Figure 7:**
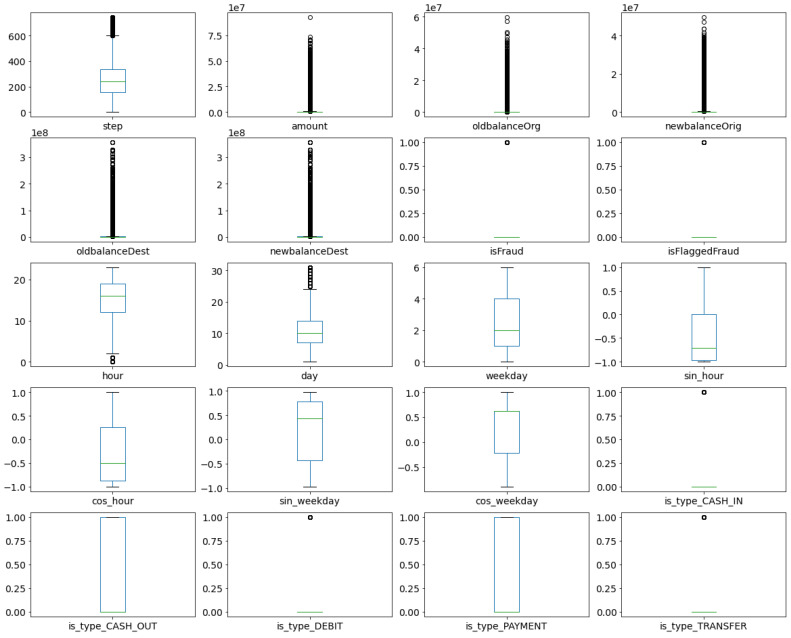
Extended *PaySim* dataset box plots.

The following experiments were conducted on the extended *PaySim* dataset, consisting of 6,362,620 entries, and 23 features. Box plots of numerical features in the extended dataset are presented in Figure 7. The box plots show that the number of outliers for nearly all original dataset features are really high. This indicates that a contamination factor used in the later methods should not be too low.

Based on box plots, it can also be concluded that none of the features is symmetrically distributed, and that the Elliptic Envelope method is not suitable for this dataset.

Additionally, box plots indicate that features amount, oldbalanceOrg, newbalanceOrig, oldbalanceDest and newbalanceDest are highly skewed, and they should be further processed before training machine learning models. The Box–Cox transformation of the SciPy package is applied to transform these features closer to a normal distribution.

##### Feature Analysis

Information Value (IV) is calculated for all features in the extended dataset in order to estimate their potential contribution to detectability.

IV of features, presented in Table 13 and Table 14, is used in the experimental part described in the following section as indicator of feature quality and reasoning for feature selection.

#### 5.2.2. Outlier Detection Approach

The results of fraud detection using selected outlier detection algorithms applied to extended *PaySim* dataset are given in this section. Similar to Case Study #1 (Section 5.1), the dataset is divided in a following manner—70% of randomly selected data in the dataset is used for training and 30% for testing. Four subsets with the same logic as in Case Study #1 were included in experiments: *S*, *S_M*, *S_M_W* and *all*.

Testing subsets of the given dataset are presented in Table 15.

The results of applying selected outlier detection methods—Local Outlier Factor (LOF) and Isolation Forest (IF) on the given subsets are presented in Table 16.

The presented results indicate that feature selection is of great importance for the dataset with this particular feature distribution as well. In this case, Local Outlier Factor is more resistant to feature selection than Isolation Forest.

**Figure 8:**
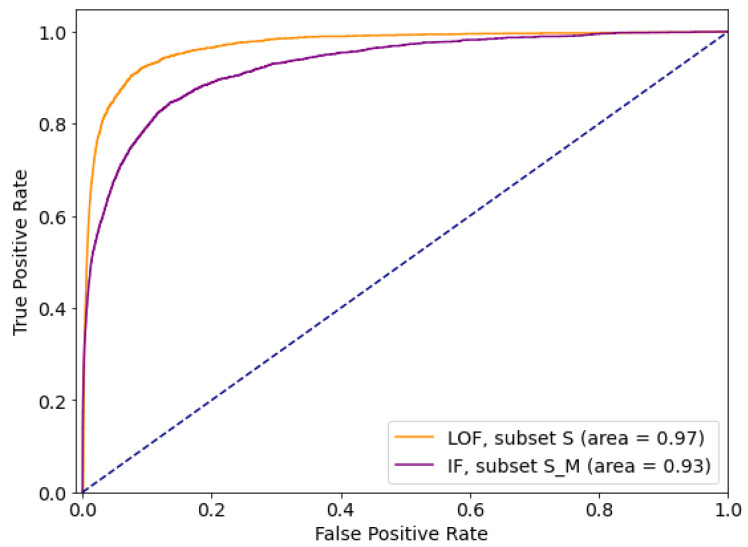
Extended *PaySim* dataset—comparative ROC curve for outlier detection methods.

Comparative ROC curves for the best performing subsets are given in Figure 8. Comparative analysis based on *tpr* and *tnr* indicates that for a given dataset Local Outlier Factor performs better, and at the same time it is resistant to feature selection, so it can be considered as the outlier detection method of choice for data with this specific feature list and distribution.

#### 5.2.3. Ensemble Approach

The results of applying ensemble methods to new extended *PaySim* dataset are given in this section. The experimental methodology from CS#1 was applied here in the same way.

The results pf applying selected ensemble methods on the given subsets are presented in Table 17.

**Figure 9:**
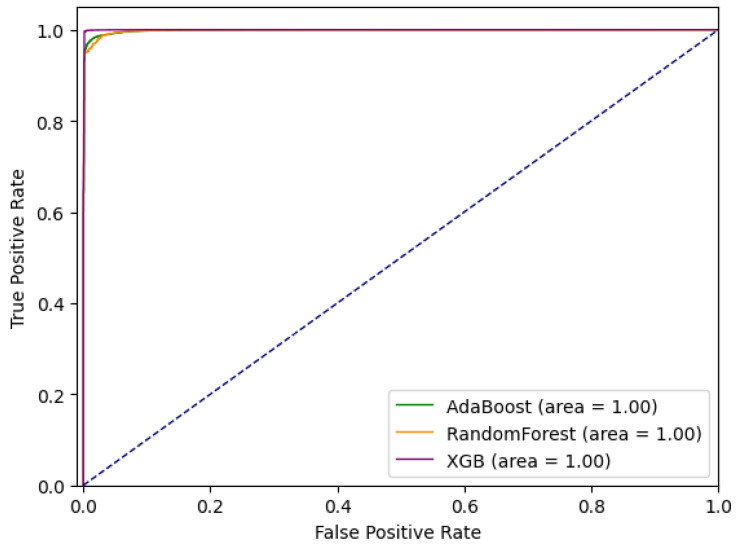
Extended *PaySim* dataset—comparative ROC curve for ensemble methods.

Comparative ROC curves for the tested ensemble methods are given in Figure 9. Comparative analysis based on *sensitivity* (*tpr*) and *specificity* (*tnr*) indicates that for a given dataset Extreme Gradient Boosting performs the best out of tested methods. At the same time, it can be noted that sensitivity is lower than specificity, and that with the cost of missing some anomalies (approximately 10%), the number of false positives is minimal. It should also be noted that ensemble approaches outperform outlier detection methods on this specific dataset.

#### 5.2.4. Reliability Analysis

##### Training of the Neural Network

The numerical features Amount, oldbalanceOrg, newbalanceOrig, oldbalanceDest and newbalanceDest were scaled to a range [0, 1]. The features step, type, nameOrig, nameDest and isFlaggedFraud were omitted.

The final dataset was split into a training, validation and test set where the test set is 20% of the whole dataset and the validation set is 10% of the training set. The total number of instances in the dataset is 6,362,620. This means that the number of instances in the training set is 4,581,086, the number of instances in the validation set is 509,010 and the number of instances in the test set is 1,272,524.

**Figure 10:**
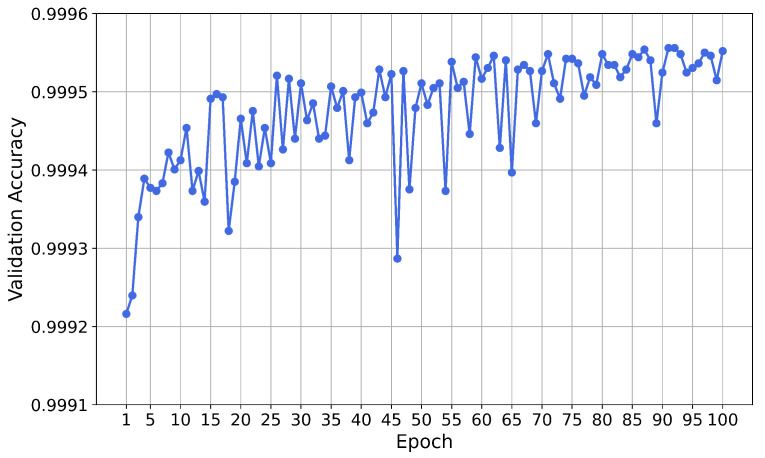
Validation accuracy of the neural network for each epoch.

The neural network was trained for 100 epochs. The validation accuracy for each epoch is shown in Figure 10. A variation of the validation accuracy of 0.0001 corresponds to an absolute variation of 51 predictions.

After the training phase, the performance of the neural network on the test set was evaluated. The accuracy on the test set is 0.9995, meaning that the network misclassified 636 instances out of 1,272,524. The ratio of fraudulent transactions which were correctly classified as fraud (true positive rate TPR) is 0.7079 and the ratio of non-fraudulent transactions which were correctly classified as non-fraud (true negative rate TNR) 0.9999.

##### Results

**Figure 11:**
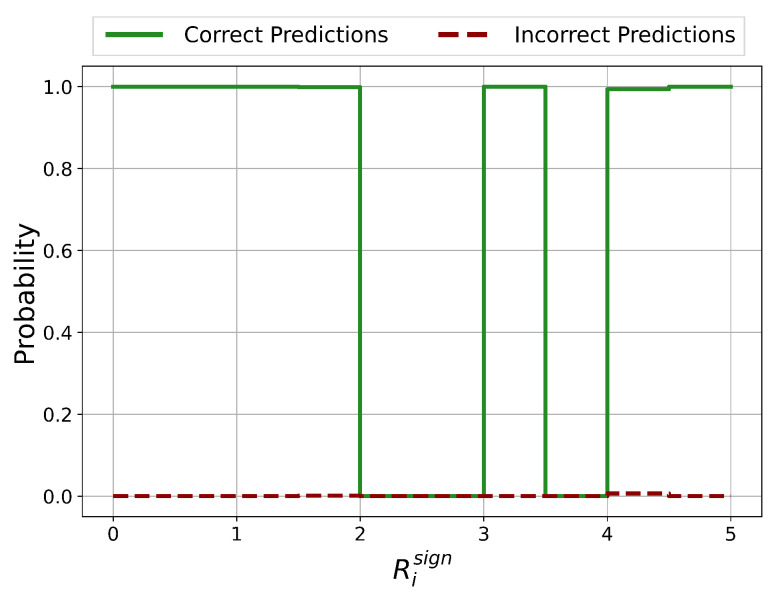
Probability distribution of ratios between the number of negative Relevance scores and the number of positive Relevance scores for correct (green solid line) and incorrect (red dashed line) predictions. For all existing values of $R_{i}^{\mathrm{sign}}$, the probability of the prediction being correct is practically 1.

The probability distributions of ratios $R_{i}^{\mathrm{sign}}$ between the number of negative and positive Relevance scores on the test set is shown in Figure 11. If the number of positive Relevance scores is zero, the ratio is set equal to the number of features (=5).

The probability of incorrect predictions is practically zero for all existing values of $R_{i}^{\mathrm{sign}}$. Values with $2<R_{i}^{\mathrm{sign}}<3$ and $3.5<R_{i}^{\mathrm{sign}}<4.0$ do not occur. This means for this dataset that $R_{i}^{\mathrm{sign}}$ is not suitable for the identification of potentially unreliable predictions. For all existing values of $R_{i}^{\mathrm{sign}}$, the probability of the prediction being correct is practically 1.

**Figure 12:**
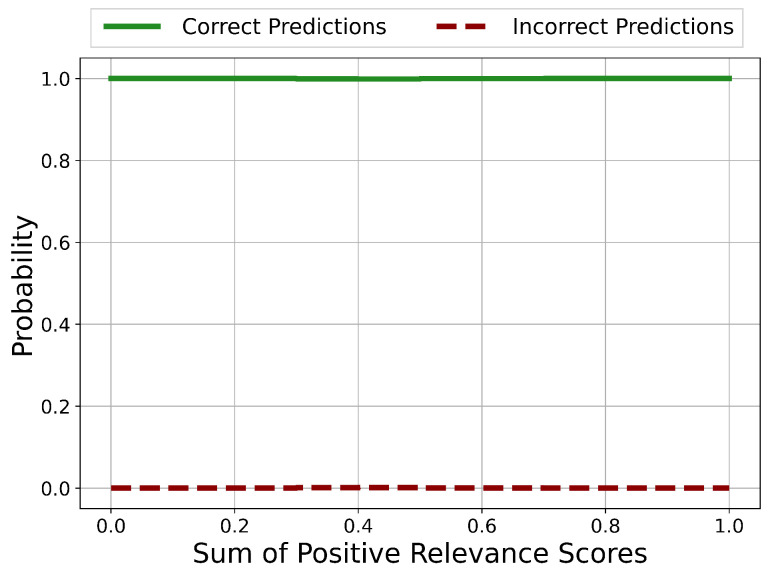
Probability distribution of the sum of positive Relevance scores for correct (green solid line) and incorrect (red dashed line) predictions on the test set. For each value, the probability of the prediction being incorrect is practically zero.

The probability distributions of the sums of positive Relevance scores in the case for correct and incorrect predictions is shown in Figure 12. For each value, the probability of the prediction being incorrect is practically zero. In this case, the sum of positive Relevance scores is not suited for the identification of incorrect predictions.

For this dataset the chosen Relevance quantities $R_{i}^{\mathrm{sign}}$ and the sum of positive Relevance scores are not suited for the identification of areas with potentially unreliable predictions. This already shows that the chosen quantities are incomplete in the sense that there are datasets for which the chosen quantities have no discriminative power.

### 5.3. CS#3: Bank Transactions Fraud Detection—BankSim Dataset

#### 5.3.1. Feature Engineering and Selection

##### Feature Engineering and Dataset Visualisation

As previously explained, *BankSim* dataset contains 10 features, representing financial transactions, namely step, customer, age, gender, zipcodeOri, merchant, zipMerchant, category, amount, fraud.

Dataset entry (financial transaction) class is annotated with feature fraud, where 0 presents “normal” transactions, and 1 presents “fraudulent” transactions. Dataset contamination is 0.21%, making the dataset highly unbalanced.

Dataset spans over six months, with the feature step presenting number of days between each transaction and the first transaction in the dataset. As such, similar to previous case studies, it does not reflect directly enough useful information, and it is a good candidate for additional feature engineering. The original feature list is extended with seven features engineered and encoded from the step feature.

There are three directly encoded features:

- month—presenting the month where the feature range is 1–6
- day—presenting the day in the month in 30 days month cycle, where the feature range is 1–30
- weekday—presenting the day in the week in seven-day week cycle, where the feature range is 1–7

These features, and especially day and weekday, do not reflect cyclic nature of time. To solve this, four new features are created deriving a sine transform and cosine transform of the respective features—sin_day, cos_day, sin_weekday and cos_weekday. The sine and cosine functions are called after normalising initial features between 0 and 2π, similar to Equations (7) and (8).

In addition to engineered time features, categorical features gender and category, containing 4 and 15 different categories, respectively, are encoded using One Hot encoding technique, adding additional 19 binary features to the dataset—is_gender_E, is_gender_F, is_gender_M, is_gender_U, and 15 similarly constructed features based on category.

**Figure 13:**
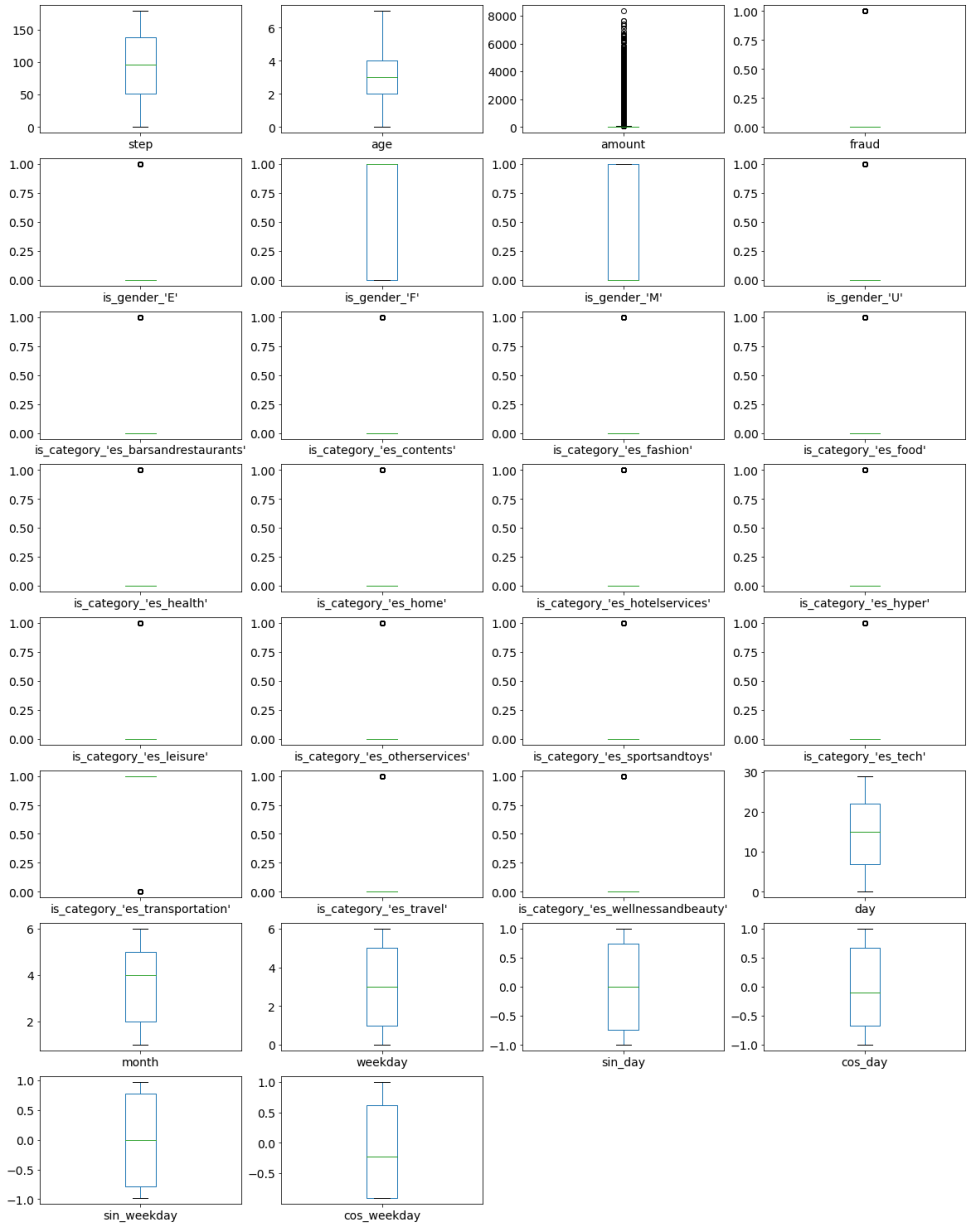
Extended *BankSim* dataset box plots.

The following experiments were conducted on extended *BankSim* dataset, consisting of 594,643 entries and 36 features. Box plots of numerical features in extended dataset are presented in Figure 13. The box plots show that the number of outliers for nearly all original dataset features are really high. This indicates that a contamination factor used in the later methods should not be too low.

The conclusion from box plots that some of the features are symmetrically distributed indicates that methods such as Elliptic Envelope can potentially be applied on this dataset.

Additionally, box plots indicate that the feature amount is highly skewed, and it should be further processed before training machine learning models. The Box–Cox transformation of the SciPy package is applied to transform this feature closer to a normal distribution.

##### Feature Analysis

Information Value (IV) is calculated for all features in the extended dataset in order to estimate their potential contribution to detectability.

IV of features, presented in Table 18 and Table 19, is used in the experimental part described in the following section as indicator of feature quality and reasoning for feature selection.

#### 5.3.2. Outlier Detection Approach

The results of fraud detection using selected outlier detection algorithms applied to the extended *BankSim* dataset are given in this section. Similar to the first two case studies (Section 5.1 and Section 5.2), 70% of randomly selected data in the dataset is used for training and 30% for testing. Four subsets with the same logic as in previous case studies are included in experiments, namely *S*, *S_M*, *S_M_W* and *all*, selected in accordance with IV.

The results of applying selected outlier detection methods—Local Outlier Factor (LOF), Isolation Forest (IF) and Elliptic Envelope (EE)—on the given subsets are presented in Table 20.

The presented results indicate that feature selection is of great importance for all tested methods.

**Figure 14:**
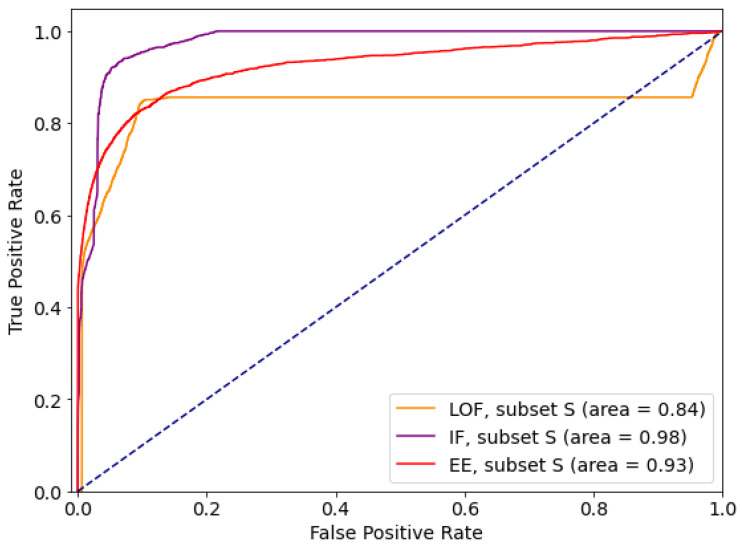
Extended *BankSim* dataset—comparative ROC curve for outlier detection methods.

Comparative ROC curves for the best performing subset are given in Figure 14. Comparative analysis based on *tpr* and *tnr* indicates that for the given dataset Isolation Forest performs the best out of tested outlier detection methods. At the same time, IF is the most resistant to feature selection, from the tested methods, so it can be considered as the outlier detection method of choice for data with this feature list and distribution.

#### 5.3.3. Ensemble Approach

The results of applying ensemble methods to new extended *BankSim* dataset are given in this section. Experiments methodology from previous case studies is applied here in the same way.

The results of applying selected ensemble methods on the given subsets are presented in Table 21.

**Figure 15:**
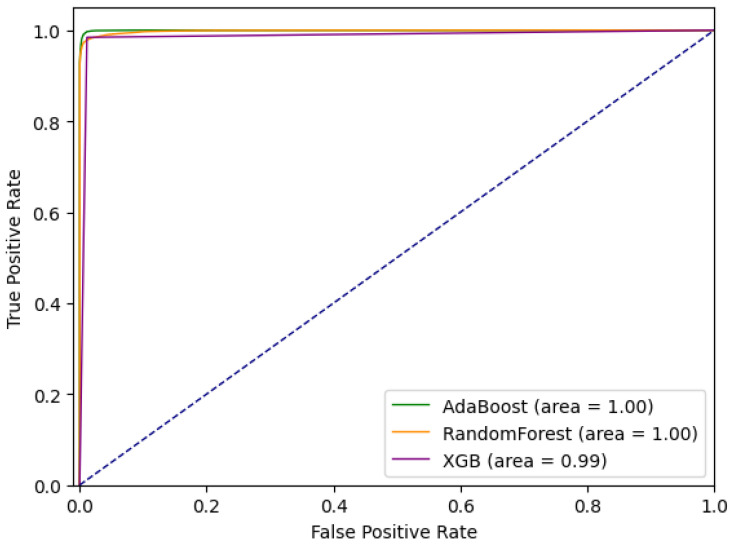
Extended *BankSim* dataset—comparative ROC curve for ensemble methods.

Comparative ROC curves for the tested ensemble methods are given in Figure 15. Comparative analysis based on *tpr* and *tnr* indicates that for a given dataset AdaBoost performs the best out of the tested methods. At the same time, it can be noted that both sensitivity and specificity are really high for all three tested methods, almost 1, which indicates that variability of features in a synthetically created dataset might not be on a high enough level. It should also be noted that ensemble approaches outperform outlier detection methods on this specific dataset.

#### 5.3.4. Reliability Analysis

##### Training of the Neural Network

The features step, customer, zipcodeOri and zipMerchant were omitted. The categorical features gender, merchant and category were converted into one-hot encoded feature vectors. The features amount and age were scaled to the range [0, 1].

The final dataset was split into a training, validation and test set where the test set is 20% of the whole dataset and the validation set is 10% of the training set. The total number of instances in the dataset is 594,643. This means that the number of instances in the training set is 428,143, the number of instances in the validation set is 47,571 and the number of instances in the test set is 118,929.

**Figure 16:**
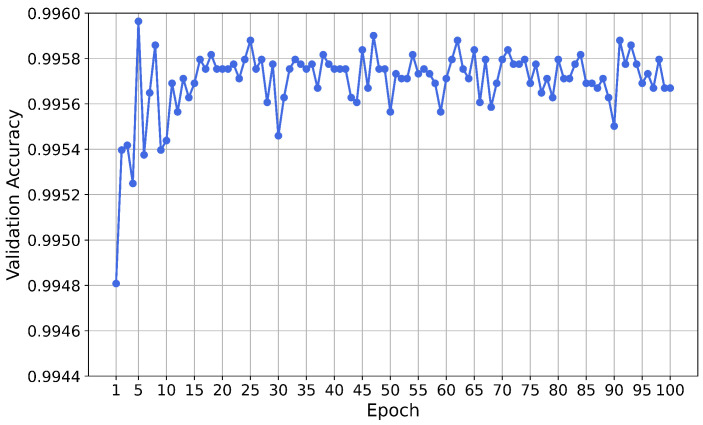
Validation accuracy of the neural network for each epoch.

The neural network was trained for 100 epochs. The validation accuracy for each epoch is shown in Figure 16. A variation of the validation accuracy of 0.0002 corresponds to an absolute variation of nine predictions.

After the training phase, the performance of the neural network on the test set was evaluated. The accuracy on the test set is 0.9958, meaning that the network misclassified 4995 instances out 118,929. The ratio of fraudulent transactions which were correctly classified as fraud (true positive rate TPR) is 0.7361 and the ratio of non-fraudulent transactions which were correctly classified as non-fraud (true negative rate TNR) 0.9990.

##### Results

**Figure 17:**
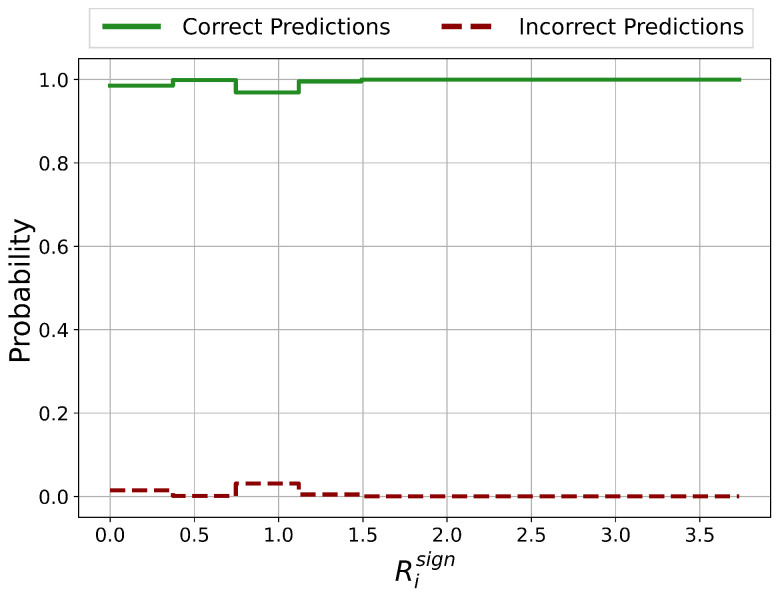
Probability distributions of ratios $R_{i}^{\mathrm{sign}}$ between the number of negative and positive Relevance scores for correct (green solid line) and incorrect (red dashed line). The predictions with $0.0<R_{i}^{\mathrm{sign}}<0.35$ and $0.75<R_{i}^{\mathrm{sign}}<1.1$ can be identified as being slightly unreliable.

The probability distributions of ratios $R_{i}^{\mathrm{sign}}$ between the number of negative and positive Relevance scores on the test set is shown in Figure 17. If the number of positive Relevance scores is zero, $R_{i}^{\mathrm{sign}}$ is set equal to the number of features (=71).

For approximately $0.0<R_{i}^{\mathrm{sign}}<0.35$ and $0.75<R_{i}^{\mathrm{sign}}<1.1$, there is a low probability of the predictions being incorrect. The predictions with values in these regions can be identified as being slightly unreliable. The probability for predictions with all other values $R_{i}^{\mathrm{sign}}$ is certain to be correct.

**Figure 18:**
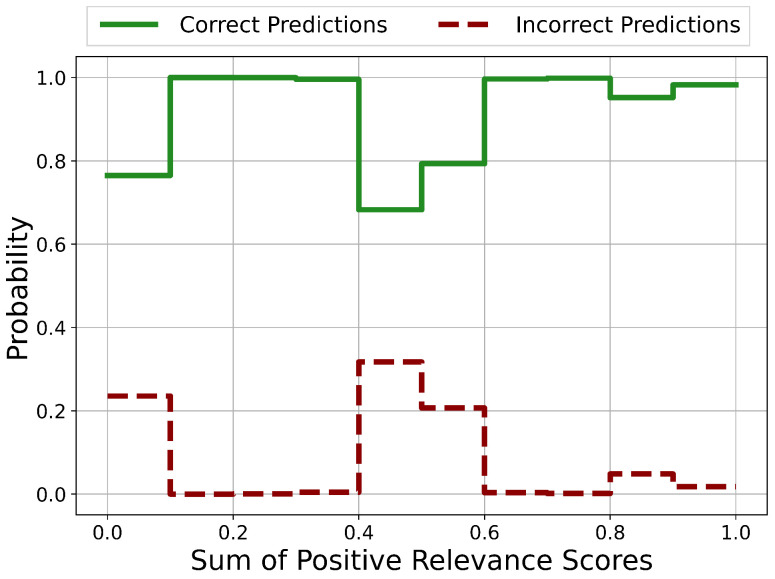
Probability distribution of the sum of positive Relevance scores for correct (green solid line) and incorrect (red dashed line) predictions on the test set.Predictions with sums of positive Relevance values with 0.0<sum<0.1 and 0.4<sum<0.6 are unreliable. Predictions with 0.8<sum<0.9 can be considered as slightly unreliable.

The probability distributions of the sums of positive Relevance scores in the case for correct and incorrect predictions is shown in Figure 18. Predictions with sums of positive Relevance values with 0.0<sum<0.1 and 0.4<sum<0.6 have a significant probability of being incorrect and therefore are unreliable. Predictions with 0.8<sum<0.9 can be considered as slightly unreliable as the probability of being incorrect is small. Predictions with other sums of positive Relevance scores can be considered as reliable as the probability of being incorrect is practically zero.

The quantity $R_{i}^{\mathrm{sign}}$ does not yield a distinction between correct and incorrect predictions as the probability of a given transaction being correct is practically 1 across all possible values of $R_{i}^{\mathrm{sign}}$. A superior quantity for the identification of potentially unreliable predictions is the sum of positive Relevance scores.

##### Reliability Analysis Results Discussion

The probability distributions of the ratio $R_{i}^{\mathrm{sign}}$ between the number of negative and positive Relevance scores does not reveal a stable indication to identify incorrect predictions across the considered datasets. Furthermore, the probability distributions of the sum of positive Relevance scores does not show stable characteristics across the three analysed datasets. For both quantities, the distributions vary significantly. However, examining a single dataset, the analysis of the probability distribution of Relevance scores can give an indication as to whether the neural network probably misclassified a specific transaction and therefore whether the predictions are reliable or not.

For CS1, predictions with $0.5<R_{i}^{\mathrm{sign}}<1.5$ and $R_{i}^{\mathrm{sign}}>3.5$ or 0.0<sum<0.2, 0.3<sum<0.4, 0.5<sum<0.6 and 0.7<sum<0.9 can be considered as unreliable in the sense that there is a significant probability that the prediction is incorrect. For CS2, there are for both quantities no regions with a significant probability of the prediction being incorrect. Therefore these quantities are in this case not suited for assessing the reliability. For CS3, predictions with 0.0<sum<0.1 and 0.4<sum<0.6 can be considered as unreliable whereas the quantity $R_{i}^{\mathrm{sign}}$ does not represent a distinctive feature for the identification of incorrect classifications.

The results of the reliability analysis with Layer-wise Relevance propagation show a variation of the probability distribution characteristics across the considered datasets. Additionally, the introduced quantities show in some cases no distinctive power. The identification of incorrect predictions across different datasets with the used quantities based on per-feature Relevance scores remains a challenging task. Therefore, a trustworthy identification of incorrect predictions probably needs more input parameters than Relevance scores alone. It is the purpose of further reliability analyses to additionally deploy other methods and to merge them into a robust metric which enables a stable reliability analysis across different datasets and machine learning algorithms.

## 6. Discussion on Implications of Fraud to Society and Call for Action

Besides the technical challenges in detecting behaviour, another aspect of the Fintech domain needs to be discussed. As already mentioned in the work of Ryman-Tubb et al. [24], financial frauds affect society in non-financial ways as well. Due to the severity of these impacts, these issues have to be addressed further. In Fintech, a fraudulent act represents a criminal offence and needs to be treated as such. However, financial crime is often regarded as minor crime and considered not to cause serious consequences. Unfortunately, this opinion is flawed and needs to be revised. In fact, financial frauds can often be linked to other major criminality such as financing of terrorism and other serious crime worldwide [106]. In this way, frauds in Fintech can represent the starting point in the chain of such criminal acts. Therefore, fraud detection represents the first line of defence in this matter.

However, significant effort needs to be made not only to block such fraud but also to identify the perpetrators. Besides detection and prevention, another dimension must be added to fraud management, namely prosecution. This challenge, however, demands engagement of all responsible authorities in the society. For this matter, a comprehensive fraud management system (FMS) [24] needs to be introduced that integrates financial institutions with the police and the criminal justice system. In this way, Fintech transactions become subject to a financial inspectorate, which eases the task for tribunals to prosecute responsible actors.

Crime syndicates usually rely on continuous financial flows, which includes suspicious sources. Since fraud is often perpetrated in repetitive patterns, some insight into the criminal’s behavioural pattern is needed. For this, the proposed anomaly detection methods can be applied to recognise such patterns. However, such a task requires the analysis to include, large-scale real-world datasets. As already mentioned, such datasets are not usually made availabale by financial institutions, so the research community has no access to them. Another problem in tracking of fraud origins is the lack of appropriate regulations for financial transactions. Unlike in other domains (e.g., [107]), conventions for transactions in online markets are often loosely defined and hard to comprehend [108]. To overcome this, transparency must be ensured for all conducted transactions by law.

With this paper, we echo the concerns of our colleagues from the research community in raising awareness of the fact that financial frauds represent a greater risk to society than currently anticipated. A systematic approach to combating such crimes requires the existing fraud detection techniques to be integrated into an existing FMS and offered to financial institutions.

## 7. Conclusions and Future Work

In this paper, we examine the contemporary security challenges in the digital world of financial transactions processing and focus on detection of anomalous (fraudulent) transactions, whereby malicious actions are conducted for illegitimate financial gain. Fraud comes in different forms that eventually cause serious consequences to the affected victims. Some ML approaches have already been successfully applied to automatically detect fraud in financial transactions. Therefore, the contribution of this paper is twofold. First, it provides a survey of existing methods from the ML domain, and publicly available datasets. In this survey, several approaches are examined, which discuss and apply intelligent solutions to identify fraudulent behaviour. The second contribution represents an evaluation of ML methods for anomaly detection. Accordingly, for this benchmarking experiment, multiple algorithms have been implemented and run, including outlier detection methods and ensemble methods, to detect fraud in financial datasets with varying success. Feature engineering and analysis has been carried out in order to estimate the influence of feature selection on detection performance.The experiments were conducted in three different case studies using various datasets and configurations. Finally, the results have been examined and the success and performance of individual methods addressed.

The results of the conducted experiments confirm the benefits of ML. Firstly, existing ML algorithms succeeded in detecting anomalies within complex datasets. In addition, the results confirm that ML methods can successfully contribute to security in Fintech systems by way of supporting enhanced fraud detection capability. Additionally, it was established that feature engineering and selection can critically influence the performance of certain algorithms, and that careful selection of features can increase overall performance and limit the negative influence of some features. It should also be noted that ensemble methods maintained more robust performance responsive to variable feature selection scenarios, performing very well in general, and in most cases better then outlier detection methods—ensemble approaches significantly outperformed outlier detection methods on the two tested synthetic datasets (*PaySim* and *BankSim*), while results on the tested dataset containing real data (*CreditCard*) are comparable for the two approaches.

However, despite the highly effective performance of the ML methods as developed and tested in this work, it has to be recognised that some challenges remain. For example, the results of the fraud detection heavily depend upon the initial configuration. The performance of various algorithms is subject to various trade-offs. For example, high detection rate of fraudulent transactions can lead to higher number of false positives. Therefore, there is still scope for improvement for the existing ML methods.

In the future, it is planned to contribute to tackling these challenges in the following ways. First, the shortcomings of the results would need to be addressed. This can be achieved by extending the initial configuration with additional parameters.The available ML algorithms on other domains will also be deployed. Unfortunately, the fact that available real-world datasets are very sparse limits the scope for further applications. Therefore, acquiring additional Fintech data would provide more insights on the applicability of existing algorithms. Fraud vectors themselves will be investigated more in detail, in order to understand the exact attack models and their indicators and the potential change in these vectors through time. The research community would benefit greatly from such work that could result in creating a new Fintech dataset that can be used as a benchmark dataset to support research in this field. Finally, the tested applications will be set up as part of a real-world anomaly detection framework, and to investigate the influence of trade-offs between the detection rate and false alerts.

## Figures and Tables

**Table 1:** Categorical variable encoding overview.

Technique	Map	Pro’s and Con’s
*One Hot Encoding*	Each category is mapped to a vector containing 1 and 0 to show presence or absence of feature.	Resulting in additional columns slowing down learning in the case that there are many different categories in a feature.
*Label Encoding*	Each category is getting a value from 1 to *N* (*N* is the number of categories).	An algorithm might consider an order indicated by the encoding which does not reflect the original categories.
*Frequency Encoding*	It uses the frequency of the categories as labels.	In the case that the frequency is related to the targeted variable, it is advantageous for the model.
*Weight of Evidence Encoding*	It is a measure to estimate its support (or the opposite) to a hypothesis.	The method was developed for building predictive models for a risk-evaluation of loan default in the credit and financial industry.
*Hashing Encoding*	Map variables to a higher dimensional space of integers.	This method pays off in the case of a high cardinality of categorical variables.
*Leave One Out Encoding*	It uses the mean of the target variable for all records (except current one).	Different encoding in training and validation/ test data.

**Table 2:** Information Value statistic rules in credit scoring overview.

Information Value	Variable Predictiveness
<0.02	Not useful for prediction
0.02–0.1	Weak predictive power
0.1–0.3	Medium predictive power
>0.3	Strong predictive power

**Table 3:** Architecture of the fully connected neural network which is used to deploy Layer-wise Relevance Propagation.

Layer	Number of Neurons	Activation Function
Input layer	23	none
Dense layer 1	500	ReLU
Dense layer 2	200	ReLU
Output layer	2	softmax

**Table 4:** Credit Card Fraud Detection dataset overview.

*Dataset name*	Credit Card Fraud Detection
*Domain*	Credit Cards
*Url*	https://www.kaggle.com/mlg-ulb/creditcardfraud (accessed on 30 November 2020)
*Year*	2013
*Type*	Real data
*Subset*	*creditcard.csv*
*Annotated*	Yes
*Unbalanced*	Yes
*No. of entries*	284,807
*Contamination rate*	0.172%
*Time duration*	2 days
*No. of features*	31
*List of features*	Time, V1, V2, V3, V4, V5, V6, V7, V8, V9, V10, V11, V12, V13, V14, V15, V16, V17, V18, V19, V20, V21, V22, V23, V24, V25, V26, V27, V28, Amount, Class

**Table 5:** Synthetic Financial Datasets for Fraud Detection dataset overview.

*Dataset name*	Synthetic Financial Datasets for Fraud Detection
*Domain*	Financial Transactions
*Url*	https://www.kaggle.com/ntnu-testimon/paysim1 (accessed on 30 November 2020)
*Year*	2015
*Type*	Synthetic data
*Subset*	*PS_20174392719_1491204439457_log.csv*
*Annotated*	Yes
*Unbalanced*	Yes
*No. of entries*	6,362,620
*Contamination rate*	0.129%
*Time duration*	1 month
*No. of features*	11
*List of features*	step, type, amount, nameOrig, oldbalanceOrg, newbalanceOrig, nameDest, oldbalanceDest, newbalanceDest, isFraud, isFlaggedFraud

**Table 6:** Synthetic data from a financial payment system dataset overview.

*Dataset name*	Synthetic data from a financial payment system
*Domain*	Financial Transactions
*Url*	https://www.kaggle.com/ntnu-testimon/banksim1 (accessed on 30 November 2020)
*Year*	2014
*Type*	Synthetic data
*Subset*	*bs140513_032310.csv*
*Annotated*	Yes
*Unbalanced*	Yes
*No. of entries*	594,643
*Contamination rate*	1.21%
*Time duration*	6 months
*No. of features*	10
*List of features*	step, customer, age, gender, zipcodeOri, merchant, zipMerchant, category, amount, fraud
*Subset*	*bsNET140513_032310.csv*
*Annotated*	Yes
*Unbalanced*	Yes
*No. of entries*	594,643
*Contamination rate*	1.21%
*Time duration*	6 months
*No. of features*	5
*List of features*	Source, Target, Weight, typeTrans, fraud

**Table 7:** Bank Transaction Data dataset overview.

*Dataset name*	Bank Transaction Data
*Domain*	Financial Transactions
*Url*	https://www.kaggle.com/apoorvwatsky/bank-transaction-data (accessed on 30 November 2020)
*Year*	2017
*Type*	Synthetic data
*Subset*	*bank.xlsx*
*Annotated*	No
*Unbalanced*	n/a
*No. of entries*	116,201
*Contamination rate*	n/a
*Time duration*	7 months
*No. of features*	8
*List of features*	Account No., Date, Transaction Details, Cheque No., Value Date, Withdrawal Amount, Deposit Amount, Balance Amount

**Table 8:** Extended *CreditCard* dataset information value—Part 1.

Strong Predictive Power			
**feature**	**IV**	**feature**	**IV**
V4	2.46	V1	0.81
V14	2.18	V21	0.75
V12	2.04	V6	0.58
V3	1.74	V27	0.56
V11	1.72	V18	0.55
V10	1.64	V28	0.54
V16	1.27	V5	0.50
V2	1.27	V8	0.42
V17	1.11	V20	0.40
V9	1.04	V19	0.38
V7	1.00	Amount	0.29

**Table 9:** Extended *CreditCard* dataset information value—Part 2.

Medium		Weak		Useless	
**feature**	**IV**	feature	**IV**	**feature**	**IV**
V23	0.14	V24	0.08	V25	0.01
sin_tot_seconds	0.12	Time	0.05	V22	0.01
tot_seconds	0.11	V26	0.03	V15	0.00
		V13	0.02		
		cos_tot_seconds	0.02		

**Table 10:** *CreditCard* fraud detection testing subsets.

*Subset*	*Features*
*S*	V4, V14, V12, V3, V11, V10, V16, V2, V17, V9, V7, V1, V21, V6, V27, V18, V28, V5, V8, V20, V19, Amount
*S_M*	V4, V14, V12, V3, V11, V10, V16, V2, V17, V9, V7, V1, V21, V6, V27, V18, V28, V5, V8, V20, V19, Amount, V23, sin_tot_seconds,tot_seconds
*S_M_W*	V4, V14, V12, V3, V11, V10, V16, V2, V17, V9, V7, V1, V21, V6, V27, V18, V28, V5, V8, V20, V19, Amount, V23, sin_tot_seconds,tot_seconds, V24, Time, V26, V13, cos_tot_seconds
*all*	V4, V14, V12, V3, V11, V10, V16, V2, V17, V9, V7, V1, V21, V6, V27, V18, V28, V5, V8, V20, V19, Amount, V23, sin_tot_seconds,tot_seconds, V24, Time, V26, V13, cos_tot_seconds, V25, V22, V15

**Table 11:** Extended *CreditCard* dataset outlier detection based fraud detection—results.

	Local Outlier Factor		Isolation Forest		Elliptic Envelope	
	*tpr*	*tnr*	*tpr*	*tnr*	*tpr*	*tnr*
*S*	0.4412	0.9008	0.9265	0.8992	0.8824	0.9003
*S_M*	0.8824	0.8960	0.9118	0.9001	0.7500	0.8994
*S_M_W*	0.7574	0.8810	0.8971	0.8996	0.8456	0.9002
*all*	0.7426	0.8814	0.9044	0.9001	0.8824	0.9002

**Table 12:** Extended *CreditCard* dataset ensemble methods based fraud detection—results.

	Random Forest		Adaptive Boosting		Extreme Gradient Boosting	
	*tpr*	*tnr*	*tpr*	*tnr*	*tpr*	*tnr*
*all*	0.7959	0.9999	0.7959	0.9998	0.8163	0.9999

**Table 13:** Extended *PaySim* dataset information value—Part 1.

Strong Predictive Power			
feature	**IV**	feature	**IV**
nameDest	3.21	amount	0.76
oldbalanceOrg	2.09	sin_hour	0.32
newbalanceOrig	1.01	day	0.30
is_type_TRANSFER	0.99	step	0.28
type	0.79		

**Table 14:** Extended *PaySim* dataset information value—part 2.

Medium		Weak		Useless	
feature	**IV**	feature	**IV**	feature	**IV**
hour	0.22	is_type_CASH_IN	0.05	newbalanceDest	0.00
oldbalanceDest	0.18	cos_weekday	0.05	is_type_DEBIT	0.00
cos_hour	0.18	weekday	0.05	isFlaggedFraud	0.00
is_type_PAYMENT	0.14	sin_weekday	0.03		
is_type_CASH_OUT	0.09	nameOrig	0.02		

**Table 15:** *PaySim* fraud detection testing subsets.

*Subset*	*Features*
*S*	oldbalanceOrg, newbalanceOrig, is_type_TRANSFER, amount, sin_hour, day, step
*S_M*	oldbalanceOrg, newbalanceOrig, is_type_TRANSFER, amount, sin_hour, day, step, hour, oldbalanceDest, cos_hour, is_type_PAYMENT, is_type_CASH_OUT
*S_M_W*	oldbalanceOrg, newbalanceOrig, is_type_TRANSFER, amount, sin_hour, day, step, hour, oldbalanceDest, cos_hour, is_type_PAYMENT, is_type_CASH_OUT, is_type_CASH_IN, cos_weekday, weekday, sin_weekday
*all*	oldbalanceOrg, newbalanceOrig, is_type_TRANSFER, amount, sin_hour, day, step, hour, oldbalanceDest, cos_hour, is_type_PAYMENT, is_type_CASH_OUT, is_type_CASH_IN, cos_weekday, weekday, sin_weekday, newbalanceDest, is_type_DEBIT, isFlaggedFraud

**Table 16:** Extended *PaySim* dataset outlier detection based fraud detection—results.

	Local Outlier Factor		Isolation Forest	
	*tpr*	*tnr*	*tpr*	*tnr*
*S*	0.9326	0.8875	0.8283	0.8103
*S_M*	0.9248	0.8923	0.8838	0.8099
*S_M_W*	0.9240	0.8923	0.8456	0.8102
*all*	0.9257	0.8933	0.8078	0.8096

**Table 17:** Extended *PaySim* dataset ensemble methods based fraud detection—results.

	Random Forest		Adaptive Boosting		Extreme Gradient Boosting	
	*tpr*	*tnr*	*tpr*	*tnr*	*tpr*	*tnr*
*all*	0.9761	0.9793	0.7171	0.9999	0.8856	1.000

**Table 18:** Extended *BankSim* dataset information value—Part 1.

Strong		Medium		Weak	
feature	IV	feature	IV	feature	IV
merchant	4.47	is_category_wellnessandbeauty	0.11	is_gender_F	0.06
customer	2.54	is_category_home	0.11	is_gender_M	0.05
amount	2.14	is_category_otherservices	0.10	is_category_hyper	0.04
is_category_transportation	1.69			is_category_tech	0.03
is_category_sportsandtoys	1.28				
is_category_health	0.53				
is_category_leisure	0.49				
is_category_travel	0.47				
is_category_hotelservices	0.28				

**Table 19:** Extended *BankSim* dataset information value—Part 2.

Useless Predictive Power			
feature	**IV**	feature	**IV**
step	0.01	cos_day	0.00
month	0.01	sin_weekday	0.00
age	0.01	weekday	0.00
is_category_barsandrestaurants	0.00	is_category_contents	0.00
is_category_food	0.00	is_gender_U	0.00
is_category_fashion	0.00	cos_weekday	0.00
is_gender_E	0.00	zipMerchant	0.00
day	0.00	zipcodeOri	0.00
sin_day	0.00		

**Table 20:** Extended *BankSim* dataset outlier detection based fraud detection—results.

	Local Outlier Factor		Isolation Forest		Elliptic Envelope	
	*tpr*	*tnr*	*tpr*	*tnr*	*tpr*	*tnr*
*S*	0.8511	0.8908	0.9546	0.9005	0.8299	0.8998
*S_M*	0.8681	0.8891	0.9787	0.9002	0.8242	0.8995
*S_M_W*	0.8006	0.8862	0.9849	0.9005	0.8166	0.8999
*all*	0.2911	0.8989	0.5803	0.8992	0.3653	0.8994

**Table 21:** Extended *BankSim* dataset ensemble methods based fraud detection—results.

	Random Forest		Adaptive Boosting		Extreme Gradient Boosting	
	*tpr*	*tnr*	*tpr*	*tnr*	*tpr*	*tnr*
*all*	0.9956	0.9673	0.9931	0.9915	0.9885	0.9843

## Data Availability

Not applicable.
